# A checklist of chromosome numbers and a review of karyotype variation in Odonata of the world

**DOI:** 10.3897/CompCytogen.v14i4.57062

**Published:** 2020-10-22

**Authors:** Valentina G. Kuznetsova, Natalia V. Golub

**Affiliations:** 1 Department of Karyosystematics, Zoological Institute, Russian Academy of Sciences, Universitetskaya emb. 1, St. Petersburg 199034, Russia Zoological Institute, Russian Academy of Sciences St. Petersburg Russia

**Keywords:** Chromosome numbers, damseldragons, damselflies, dragonflies, m-chromosomes, sex chromosome mechanisms

## Abstract

The ancient insect order Odonata is divided into three suborders: Anisoptera and Zygoptera with approximately 3000 species worldwide each, and Anisozygoptera with only four extant species in the relict family Epiophlebiidae. An updated list of Odonata species studied regarding chromosome number, sex chromosome mechanism and the occurrence of m-chromosomes (= microchromosomes) is given. Karyotypes of 607 species (198 genera, 23 families), covering approximately 10% of described species, are reported: 423 species (125 genera, 8 families) of the Anisoptera, 184 species (72 genera, 14 families) of the Zygoptera, and one species of the Anisozygoptera. Among the Odonata, sex determination mechanisms in males can be of X(0), XY and X_1_X_2_Y types, and diploid chromosome numbers can vary from 6 to 41, with a clear mode at 2n = 25(60%) and two more local modes at 2n = 27(21%) and 2n = 23(13%). The karyotype 2n = 25(24A + X) is found in each of the three suborders and is the most typical (modal) in many families, including the best-covered Libellulidae, Corduliidae (Anisoptera), Lestidae, Calopterygidae, and Platycnemididae (Zygoptera). This chromosome set is considered ancestral for the Odonata in general. Chromosome rearrangements, among which fusions and fissions most likely predominated, led to independent origins of similar karyotypes within different phylogenetic lineages of the order. The karyotype 2n = 27(26A + X) prevails in Aeshnidae and Coenagrionidae, whereas the karyotype 2n = 23(22A + X) is modal in Gomphidae and Chlorocyphidae, in both pairs of families one being from the Anisoptera while the other from the Zygoptera.

## Introduction

The order Odonata, which comprises slightly more than 6,000 described species worldwide, is one of the most ancient among winged insects (Pterygota), dating from the Permian (Grimaldi and Engel 2005). Extant Odonata include two main suborders with approximately 3,000 species each, the Zygoptera or damselflies with about 308 genera and the Anisoptera or true dragonflies with about 344 genera. Within these suborders, up to 21 and 11 families (and sometimes more), respectively, are currently recognized. The third suborder, the Anisozygoptera or damseldragons, includes only one genus *Epiophlebia* Calvert, 1903 with four extant species in the relict family Epiophlebiidae. A substantial body of evidence indicates that Anisoptera and Zygoptera are each monophyletic, and Zygoptera are sister to *Epiophlebia* plus Anisoptera (Rehn 2003; Kalkman et al. 2008; Dijkstra et al. 2013, 2014; Schorr and Paulson 2020).

**Figure 1.**
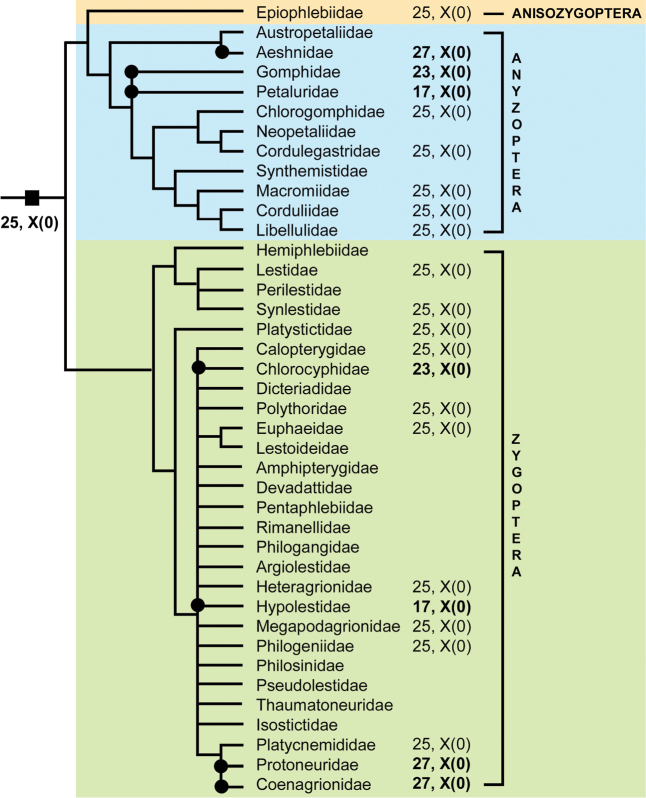
Mapping of modal karyotypes onto phylogenetic tree of Odonata families. The phylogenetic tree is taken from Bybee et al. (2016) who synthesized it based on trees from Dijkstra et al. (2014) and Carle et al. (2015). Plesiomorphic karyotype state is indicated by a black solid **square** (■), apomorphic karyotype states are indicated by black solid **circles** (●).

The field of Odonata cytogenetics was heavily influenced by Bastiaan Kiauta, who has published dozens of papers and analyzed karyotypes of about 260 species and subspecies of this group (see References and Table 1). During the years that have passed since the publication of chromosome number checklist of Odonata (Kiauta 1972c), approximately 90 chromosome papers have been published. The number of examined species has since increased by more than 2.3 times, and now it seems appropriate to publish an updated list. In this review article, all data available today are presented in two tables and one figure. Table 1 includes all species studied so far cytogenetically and compiles data on their chromosome numbers, sex chromosome mechanisms and the occurrence of the so-called m-chromosomes (= microchromosomes). Table 2 summarizes data presented in Table 1 and shows the family-level variability of the above-mentioned traits (except m-chromosomes, since data on their presence or absence in specific species are often questionable) together with the most characteristic (modal) karyotypes for each of the families explored. On the Fig. 1, the modal karyotypes are mapped onto phylogenetic tree of Odonata families taken from Bybee et al. (2016) who in turn redrawn and synthesized it from Dijkstra et al. (2014) and Carle et al. (2015). In the final section of the review, the main characteristics of Odonata karyotypes are briefly discussed and prospects for future research are outlined.

## Concluding remarks and future directions

In total, karyotypes of 607 species (198 genera, 23 families) of Odonata are studied up to now. Table 1, presented in our work, includes 423 species (125 genera, 8 families) of the Anisoptera, 184 species (72 genera, 14 families) of the Zygoptera, and one species of the Anisozygoptera. Thus, the presently available karyotype data cover about 10% of the world species diversity of the order in general.

**Table 1.** Cytogenetically analyzed species of Odonata and their main karyotype characteristics (chromosome numbers, sex chromosomes, m-chromosomes).

Taxon		Karyotype formula 2n ♂	m-chromo somes	Country	References
** Anisozygoptera **					
** Epiophlebioidea **					
** Epiophlebiidae **					
1.	***Epiophlebia****superstes* Selys, 1889	25(24A+X)	–	Japan	Oguma 1951
** Anisoptera **					
** Aeshnoidea **					
** Aeshnidae **					
2.	***Aeshna****caerulea* (Ström, 1783)	24(22A+neo-XY)	–	Finland	Oksala 1943
3.	*A. canadiensis* Walker, 1908	27(26A+X)	+	USA	Cruden 1968
4.	*A. clepsydra* Say, 1839	27(26A+X)	+	USA	Hung 1971
5.	*A. crenata* Hagen, 1856	27(26A+X)	+	Finland	Oksala 1939a, 1943, 1944, 1952
		– » –	–	Russia	Perepelov and Bugrov 2002
6.	*A. cyanea* (Müller, 1764)	27(26A+X)	+	Finland	Oksala 1943
		– » –	+	Netherlands	Kiauta 1969a
7.	*A. grandis* (Linnaeus, 1758)	27(26A+X)	+	Former USSR	Fuchsówna and Sawczyńska 1928
		25(24A+X)	+	Former USSR	Makalowskaja 1940
		26(24A+neo-XY)	+	Finland	Oksala 1939a, 1943, 1944, 1945
		– » –	+	Netherlands	Kiauta 1967a–[Bibr B44][Bibr B46], b, 1969a
		– » –	+	Russia	Perepelov and Bugrov 2002
		25(24A+X)	–	Finland	Nokkala et al. 2002
8.	*A. isoceles* (Müller, 1767)	27(26A+X)	–	USA	Kiauta 1978 as *Anaciaeschna isosceles* (Müller, 1767)
		25(24A + X)	+	Russia	Kuznetsova et al. 2020b
9.	*A. juncea* (Linnaeus, 1758)	26(24A+neo-XY)	+	Finland	Oksala 1939a, 1943, 1944
		– » –	+	Former USSR	Makalowskaja 1940
		27(26A+X)	+	Italy	Kiauta 1971a
		26(24A+neo-XY)	+	Russia	Perepelov and Bugrov 2002
10.	*A. mixta* Latreille, 1805	27(26A+X)	+	Netherlands	Kiauta 1969a
		25(24A+X)	+	India	Sandhu and Malhotra 1994a
		– » –	+	India	Sharma and Durani 1995
		27(26A+X)	+	Russia	Perepelov and Bugrov 2001b
11.	*A. nigroflava* Martin, 1909	27(26A+X)	+	Japan	Katatani 1987
		– » –	–	Russia	Perepelov and Bugrov 2002
12.	*A. palmata* Hagen, 1856	27(26A+X)	+	USA	Cruden 1968
13.	*A. serrata* Hagen, 1856	26(24A+neo-XY)	+	Finland	Oksala 1943 as *A. osiliensis* Mierzejewski, 1913 and *A. s. fennica* Valle, 1938
14.	*A. subarctica* Walker, 1908	27(26A+X)	+	USA	Oksala 1939a, 1943, 1952 as *A. s. elisabethae* Djakonov, 1922
		– » –	+	Switzerland	Kiauta and Kiauta 1980a as *A. s. elisabethae*
15.	*A. umbrosa* Walker, 1908	27(26A+X)	+	USA	Cruden 1968 as *A. u. occidentalis* Walker, 1908 and *A. u. umbrosa* Walker, 1908
16.	*A. verticalis* Hagen, 1861	27(26A+X)	+	USA	Hung 1971
17.	*A. viridis* Eversmann, 1836	26(24A+neo-XY)	+	Finland	Oksala 1943
		– » –	+	Russia	Perepelov et al. 1998
18.	*A. walkeri* Kennedy, 1917	27(26A+X)	+	USA	Cruden 1968
19.	***Anaciaeschna****jaspidea* (Burmeister, 1839)	27(26A+X)	+	India	Walia and Sandhu 1999
20.	***Anax****amazili* (Burmeister, 1839)	27(26A+X)	–	Argentina	Capitulo et al. 1991
		– » –	+	Argentina	Mola et al. 1999
21.	*A. concolor* Brauer, 1865	27(26A+X)	+	Surinam	Kiauta 1979a
22.	*A. ephippiger* (Burmeister, 1839)	13(12A+X)	+	India	Seshachar and Bagga 1962 as *Hemianax ephippiger* (Burmeister, 1839)
		14(12A+neo-XY)	+	India	Kiauta 1969a as *H. ephippiger*
23.	*A. guttatus* (Burmeister, 1839)	15(14A+X)	+	Nepal	Kiauta and Kiauta 1982
24.	*A. immaculiformis* Rambur, 1842	27(26A+X)	+	India	Sangal and Tyagi 1982
		– » –	+	India	Walia et al. 2018
25.	*A. imperator* Leach, 1815	27(26A+X)	+	France	Kiauta 1965, 1969a
		– » –	–	Kenya	Wasschner 1985
		– » –	+	Russia	Perepelov and Bugrov 2002
26.	*A. junius* (Drury, 1773)	27(26A+X)	+	USA	McGill 1904, 1907
		– » –	+	USA	Lefevre and McGill 1908
		– » –	–	Japan	Kichijo 1942a
		– » –	+	USA	Cruden 1968
		– » –	–		
27.	*A. longipes* Hagen, 1861	27(26A+X)	+	USA	Cruden 1968
28.	*A. nigrofasciatus* Oguma, 1915	27(26A+X)	+	Nepal	Kiauta 1974, 1975 (*A. n. nigrolineatus* Fraser, 1935)
		25(24A+X)	+	India	Sandhu and Malhotra 1994a (*A. n. nigrolineatus*)
		27(26A+X)	+	India	Walia and Sandhu 1999 (*A. n. nigrolineatus*)
		– » –	+	India	Walia et al. 2018 (*A. n. nigrolineatus*)
29.	*A. papuensis* (Burmeister, 1839)	27(26A+X)	+	Australia	Kiauta 1968c, 1969a as *Hemianax papuensis* (Burmeister, 1839)
30.	*A. parthenope* (Selys, 1839)	27(26A+X)	+	Japan	Omura 1957 as *A. parthenope julius* Brauer, 1865
		– » –	+	India	Thomas and Prasad 1986
		– » –	+	China	Zhu and Wu 1986 as *A. p. julius*
		25(24A+X)	+	Japan	Suzuki and Saitoh 1990 as *A. p. julius*
		27(26A+X)	+	India	Sandhu and Malhotra 1994a
31.	***Andaeschna****unicolor* (Martin, 1908)	27(26A+X)	+	Bolivia	Cumming 1964 as Aeshna cf. unicolor Martin, 1908
32.	***Austroaeschna****anacantha* Tillyard, 1908	27(26A+X)	+	Australia	Kiauta 1968c as *Acanthaeschna anacantha* (Tillyard, 1908)
33.	*A. multipunctata* (Martin, 1901)	27(26A+X)	+	Australia	Kiauta 1968c as *Acanthaeschna multipunctata* (Martin, 1901)
34.	***Basiaeschna****janata* (Say, 1939)	25(24A+X)	–	USA	Cruden 1968
35.	***Boyeria****maclachlani* (Selys, 1883)	27(26A+X)	+	Japan	Omura 1957
36.	*B. vinosa* (Say, 1839)	27(26A+X)	–	USA	Cruden 1968
37.	***Caliaeschna****microstigma* (Schneider, 1845)	16(14A+neo-XY)	+	Greece	Kiauta 1972a
38.	***Castoraeschna****castor* (Brauer, 1865)	27(26A+X)	+	Brazil	Kiauta 1972b
39.	***Cephalaeschna****orbifrons* Selys, 1883	25(24A+X)	+	Nepal	Kiauta 1975
40.	*Cephalaeschna* sp.	25(24A+X)	+	India	Sandhu and Malhotra 1994a
41.	***Coryphaeschna****adnexa* (Hagen, 1961)	27(26A+X)	–	Bolivia	Cumming 1964
42.	*C. perrensi* (McLachlan, 1887)	25(24A+X)	–	Argentina	Capitulo et al. 1991
		27(26A+X)	+	Argentina	Mola et al. 1999
		– » –	+	Argentina	De Gennaro et al. 2008
43.	*C. viriditas* Calvert, 1952	23(22A+X)	+	Surinam	Kiauta 1979a
44.	***Gynacantha****bayadera* Selys, 1891	25(24A+X)	+	India	Walia 2007 as *G. milliardi* Fraser, 1936
		27(26A+X)	+		
45.	*G. hyalina* Selys, 1882	28(26A+XX)*	+	India	Tyagi 1978a, b
46.	*G. interioris* Williamson, 1923	26(24A+neo-XY)	+	Surinam	Kiauta 1979a
		– » –	+	Brazil	Ferreira et al. 1979
47.	*G. japonica* Bartenev, 1909	27(26A+X)	+	Japan	Omura 1957
48.	***Gynacanthaeschna****sikkima* (Karsch, 1891)	27(26A+X)	+	India	Walia et al. 2016
49.	***Oplonaeschna****armata* (Hagen, 1861)	27(26A+X)	+	Mexico	Kiauta 1970a
50.	***Planaeschna****milnei* (Selys, 1883)	27(26A+X)	+	Japan	Kiauta 1968c, 1969a
51.	***Remartinia****luteipennis* (Burmeister, 1839)	25(24A+X)	+	Surinam	Kiauta 1979a as *Coryphaeschna l. luteipennis* Burmeister, 1839
		27(26A+X)	+	Brazil	Ferreira et al. 1979 as *C. l. luteipennis*
52.	***Rhionaeschna****bonariensis* (Rambur, 1842)	26(24A+neo-XY)	+	Argentina, Uruguay	Mola and Papeschi 1994 as *Aeschna bonariensis* Rambur, 1842
52.	***Rhionaeschna****bonariensis* (Rambur, 1842)	– » –	+	Argentina, Uruguay	Mola 1995 as *A. bonariensis*
53.	*Rh. californica* (Calvert, 1895)	27(26A+X)	+	Canada	Kiauta 1973a as *Aeshna californica* Calvert, 1895
54.	*Rh. confusa* (Rambur, 1842)	27(26A+X)	+	Argentina, Uruguay	Mola and Papeschi 1994 as *Aeshna confuse* Rambur, 1842
		– » –	+	Argentina, Uruguay	Mola 1995 as *A. confuse*
55.	*Rh. diffinis* (Rambur, 1842)	21(20A+X)	+	Bolivia	Cumming 1964 as *Aeshna d. diffinis* Rambur, 1842
56.	*Rh. intricata* (Martin, 1908)	19(18A+X)	+	Bolivia	Cumming 1964 as *Aeshna intricata* Martin, 1908
57.	*Rh. peralta* (Ris, 1918)	27(26A+X)	+	Bolivia	Cumming 1964 as *Aeshna peralta* Ris, 1918
58.	*Rh. planaltica* (Calvert, 1845)	16(14A+neo-XY)	+	Argentina	Mola and Papeschi 1994 as *Aeschna cornigera planaltica* Calvert, 1952
		– » –	+	Argentina	Mola 1995 as *A. c. planaltica*
59.	***Staurophlebia****reticulata* (Burmeister, 1839)	27(26A+X)	+	Brazil	Souza Bueno 1982 (*S. r. reticulata* (Burmeister, 1839))
** Petaluroidea **					
** Petaluridae **					
60.	***Tachopteryx****thoreyi* (Hagen, 1857)	19(18A+X)	+	USA	Cumming 1964
61.	***Tanypteryx****hageni* (Selys, 1879)	17(16A+X)	+	USA	Cruden 1968
62.	*T. pryeri* (Selys, 1889)	17(16A+X)	+	Japan	Kichijo 1939, 1942a
63.	***Uropetala****carovei* (White, 1846)	17(16A+X)**	+	New Zealand	Wolfe 1953
		25(24A+X)	+	New Zealand	Jensen and Mahanty 1978
		– » –	+	New Zealand	Jensen 1980
** Gomphoidea **					
** Gomphidae **					
64.	***Anisogomphus****bivittatus* (Selys, 1854)	23(22A+X)	+	India	Das 1956
		– » –	+	India	Walia and Chahal 2020
65.	*A. occipitalis* (Selys, 1854)	23(22A+X)	–	Nepal	Kiauta 1974, 1975
66.	***Aphylla****edentata* Selys, 1869	23(22A+X)	–	Bolivia	Cumming 1964
67.	*A. producta* Selys, 1854	23(22A+X)	–	Bolivia	Cumming 1964
68.	*A. theodorina* (Navas, 1933)	23(22A+X)	+	Surinam	Kiauta 1979a
		– » –	+	Brazil	Ferreira et al. 1979
69.	*A. williamsoni* (Gloyd, 1936)	23(22A+X)	+	USA	Kiauta and Brink 1978
70.	*Aphylla* sp.	23(22A+X)	+	Argentina	Mola 2007
71.	***Arigomphus****lentulus* (Needham, 1902)	23(22A+X)	–	USA	Cruden 1968 as *Gomphus lentulus* Needham, 1902
72.	*A. pallidus* (Rambur, 1842)	23(22A+X)	–	USA	Cumming 1964 as *Gomphus pallidus* Rambur, 1842
73.	*A. submedianus* (Williamson, 1914)	23(22A+X)	–	USA	Cruden 1968 as *Gomphus submedianus* Williamson, 1914
74.	***Asiagomphus****melaenops* (Selys, 1854)	23(22A+X)	+	Japan	Toyoshima and Hirai 1953 as *Gomphus melaenops* Selys, 1854
		– » –	+	Japan	Hirai 1956 as *G. melaenops*
		– » –	+	USA	Cruden 1968 as *G. melaenops*
75.	***Burmagomphus****pyramidalis* Laidlaw, 1922	23(22A+X)	+	India	Tyagi 1977
76.	***Davidius****nanus* (Selys, 1869)	23(22A+X)	–	Japan	Kichijo 1939, 1942a
77.	***Dromogomphus****spinosus* (Selys, 1854)	23(22A+X)	+	USA	Cruden 1968
78.	*D. spoliatus* (Hagen, 1857)	23(22A+X)	+	USA	Cruden 1968
79.	***Epigomphus****llama* Calvert, 1903	23(22A+X)	–	Bolivia	Cumming 1964
80.	***Erpetogomphus****designatus* Hagen, 1857	23(22A+X)	+	USA	Cumming 1964
81.	*E. diadophis* Calvert, 1905	23(22A+X)	–	USA	Cumming 1964
82.	*E. ophibolus* Calvert, 1905	23(22A+X)	+	Mexico	Kiauta 1970a
83.	***Gomphoides*** sp.	23(22A+X)	–	Bolivia	Cumming 1964
84.	***Gomphus****confraternus* Selys, 1873	23(22A+X)	+	USA	Cruden 1968
85.	*G. exilis* Selys, 1854	23(22A+X)	+	USA	Cruden 1968
		– » –	+	Canada	Kiauta 1969a
86.	*G. graslini* Rambur, 1842	12(10A+neo-neo-XY)	+	France	Kiauta 1968d, 1969a
87.	*G. pulchellus* Selys, 1840	23(22A+X)	+	France	Kiauta 1973b
88.	*G. vulgatissimus* (Linnaeus, 1758)	23(22A+X)	–	Russia	Perepelov et al. 2001
89.	***Ictinogomphus****rapax* (Rambur, 1942)	23(22A+X)	+	India	Asana and Makino 1935
		– » –	+	India	Makino 1935
		– » –	+	India	Kichijo 1942a
		– » –	+	India	Omura 1949, 1952, 1953
		– » –	+	India	Dasgupta 1957
90.	***Nepogomphus****modestus* (Selys, 1878)	23(22A+X)	–	India	Walia et al. 2006
		– » –	–	India	Walia and Chahal 2014
91.	***Nihonogomphus****ruptus* (Selys, 1858)	23(22A+X)	–	Russia	Perepelov et al. 2001
92.	*N. viridis* Oguma, 1926	23(22A+X)	+	Japan	Omura 1957
93.	***Nychogomphus****duaricus* (Fraser, 1924)	22(20A+neo-XY)	+	India	Tyagi 1977
94.	***Octogomphus****specularis* (Hagen, 1859)	23(22A+X)	–	USA	Cruden 1968
95.	***Onychogomphus****forcipatus* (Linnaeus, 1758)	25(24A+X)	–	Finland	Oksala 1945
		22(20A+neo-XY)	–	Austria	Kiauta 1969a
		25(24A+X)	–		
96.	*O. saundersii* Selys, 1854	22(20A+neo-XY)	+	India	Tyagi 1977 (*O. s. duaricus* Fraser, 1924)
97.	***Ophiogomphus****bison* Selys, 1873	23(22A+X)	–	USA	Cruden 1968
		25(24A+X)	–		
98.	*O. cecilia* (Fourcroy, 1785)	24(22A+XX)*	–	Finland	Oksala 1945
		23(22A+X)	–	Russia	Perepelov et al. 1998
		– » –	–	Russia	Perepelov and Bugrov 2001a
99.	*O. colubrinus* Selys, 1854	23(22A+X)	–	USA	Cruden 1968
100.	*O. obscurus* Bartenev, 1909	23(22A+X)	–	Russia	Perepelov and Bugrov 2001b
101.	*O. occidentalis* Hagen, 1882	23(22A+X)	–	USA	Cruden 1968
102.	*O. rupinsulensis* (Walsh, 1862)	23(22A+X)	–	USA	Cruden 1968
103.	***Phanogomphus****lividus* (Selys, 1854)	23(22A+X)	+	USA	Cruden 1968 as *Gomphus lividus* Selys, 1854
104.	*Ph. militaris* (Hagen, 1858)	23(22A+X)	–	USA	Cruden 1968 as *Gomphus militaris* Hagen, 1858
105.	*Ph. spicatus* (Selys, 1854)	23(22A+X)	+	USA	Cruden 1968 as *Gomphus spicatus* Selys, 1854
106.	***Paragomphus****lineatus* (Selys, 1850)	23(22A+X)	–	Nepal	Kiauta 1974, 1975
		– » –	–	India	Walia and Chahal 2014
107.	*P. capricornis* (Förster, 1914)	23(22A+X)	–	Thailand	Kiauta and Kiauta 1983
108.	***Phyllocycla****propinqua* Belle, 1972	21(20A+X)	–	Argentina	De Gennaro 2004
109.	*Phyllocycla* sp.	23(22A+X)	–	Bolivia	Cumming 1964
110.	*Phyllocycla* sp. 1	23(22A+X)	+	Argentina	Mola 2007
111.	*Phyllocycla* sp. 2	23(22A+X)	–	Argentina	Mola 2007
112.	***Phyllogomphoides****undulatus* (Needham, 1944)	23(22A+X)	+	Surinam	Kiauta 1979a
113.	***Progomphus****borealis* McLachlan, 1873	23(22A+X)	–	USA	Cruden 1968
114.	*P. intricatus* (Hagen, 1857)	23(22A+X)	–	Bolivia	Cumming 1964
115.	*P. obscurus* (Rambur, 1842)	23(22A+X)	–	USA	Cruden 1968
116.	*P. phyllochromus* Ris, 1918	23(22A+X)	+	Bolivia	Cumming 1964
117.	***Scalmogomphus****bistrigatus* (Hagen, 1854)	23(22A+X)	–	Nepal	Kiauta 1974, 1975 as *Onychogomphus bistrigatus* (Hagen, 1854)
118.	***Shaogomphus****postocularis* (Selys, 1869)	23(22A+X)	+	Japan	Omura 1957 as *Gomphus postocularis* Selys, 1869
		– » –	–	Russia	Perepelov et al. 2001 as *Gomphus epophtalmus* Selys, 1872
119.	***Sieboldius****albardae* Selys, 1886	23(22A+X)	+	Japan	Omura 1957
120.	***Stylogomphus****suzukii* (Matsumura, 1926)	23(22A+X)	+	Japan	Oguma 1930
		– » –	+	Japan	Kichijo 1942a
121.	***Stylurus****flavipes* (Charpentier, 1825)	23(22A+X)	+	Russia	Perepelov and Bugrov 2001b
122.	*S. plagiatus* (Selys, 1854)	23(22A+X)	+	USA	Cruden 1968 as *Gomphus plagiatus* Selys, 1854
123.	*S. scudderi* (Selys, 1873)	23(22A+X)	–	USA	Cruden 1968 as *Gomphus scudderi* Selys, 1873
124.	*S. townesi* Gloyd, 1936	22(20A+neo-XY)	–	USA	Kiauta and Brink 1978 as *Gomphus townesi* Gloyd, 1936
125.	***Temnogomphus****bivittatus* (Selys, 1854)	23(22A+X)	+	Nepal	Kiauta 1975
126.	***Trigomphus****citimus* (Needham, 1931)	21(20A+X)	+	Japan	Toyoshima and Hirai 1953 (*T. c. tabei* Asahina, 1949)
		– » –	+	Japan	Hirai 1956 (*T. c. tabei*)
127.	*T. interruptus* (Selys, 1854)	19(18A+X)	+	Japan	Oguma 1930
		– » –	+	Japan	Toyoshima and Hirai 1953
		– » –	+	Japan	Hirai 1956
		– » –	+	Japan	Omura 1957
128.	*T. melampus* (Selys, 1869)	21(20A+X)	–	Japan	Oguma 1930, 1942 as *T. unifasciatus* (Oguma 1926)
129.	***Zonophora****callipus* Selys, 1869	23(22A+X)	+	Surinam	Kiauta 1979a
** Libelluloidea **					
** Macromiidae **					
130.	***Didymops****transversa* (Say, 1839)	25(24A+X)	+	USA	Cruden 1968
131.	***Epophthalmia****frontalis* (Selys, 1871)	25(24A+X)	+	India	Dasgupta 1957 (*E. f. frontalis* (Selys, 1871))
132.	***Macromia****daimoji* Okumura, 1949	25(24A+X)	–	Japan	Katatani 1987
133.	*M. amphigenia* Selys, 1871	25(24A+X)	–	Russia	Perepelov and Bugrov 2001b (*M. a. fraenata* Martin, 1906)
134.	*M. magnifica* (McLachlan, 1874)	25(24A+X)	+	USA	Cruden 1968
		– » –	–		
135.	*M. moorei* Selys, 1874	25(24A+X)	+	Nepal	Kiauta 1977
		– » –	+	India	Walia and Chahal 2018
** Corduliidae **					
136.	***Cordulia****aenea* (Linnaeus, 1758)	25(24A+X)	–	Finland	Oksala 1939a
		– » –	–	Former USSR	Makalowskaja 1940
		– » –	–	Netherlands	Kiauta 1968b, 1969a
		– » –	–	Russia	Perepelov et al. 1998
		– » –	–	Bulgaria	Grozeva and Marinov 2007
		– » –	–	Russia	Kuznetsova et al. 2018
137.	*C. shurtleffi* Scudder, 1866	25(24A+X)	+	USA	Cruden 1968
		– » –	+	Canada	Kiauta 1973a
138.	***Dorocordulia****libera* (Selys, 1871)	11(10A+X)	–	USA	Cruden 1968
		13(12A+X)	–		
		14(12A+neo-XY)	–	USA	Kiauta 1969a
		13(12A+X)	–		
139.	***Epicordulia****princeps* (Hagen, 1861)	25(24A+X)	+	USA	Hung 1971
140.	***Epitheca****bimaculata* (Charpentier, 1825)	25(24A+X)	–	Russia	Perepelov 2003
		– » –	–	Russia	Kuznetsova et al. 2018
141.	*E. canis* McLachlan, 1886	25(24A+X)	+	USA	Cruden 1968
142.	*E. cynosura* (Say, 1839)	19(18A+X)	–	USA	Cruden 1968
		21(20A+X)	–		
143.	*E. petechialis* (Muttkowski, 1911)	21(20A+X)	–	USA	Cumming 1964 as *Tetragoneuria petechialis* Muttkowski, 1911
144.	*E. semiaquea* (Burmeister, 1839)	25(24A+X)	–	USA	Cruden 1968
145.	*E. spinigera* (Selys, 1871)	25(24A+X)	+	USA	Cruden 1968
		27(26A+X)	–	USA	Hung 1971 as *Tetragoneuria spinigera* (Selys, 1871)
146.	***Procordulia****grayi* (Selys, 1871)	25(24A+X)	+	New Zealand	Jensen 1980
147.	*P. smithii* (White, 1846)	25(24A+X)	+	New Zealand	Jensen 1980
148.	***Rialla****villosa* Rambur, 1842	25(24A+X)	+	Argentina	De Gennaro 2004
149.	***Somatochlora****alpestris* (Selys, 1840)	25(24A+X)	–	Switzerland	Kiauta and Kiauta 1980a
		27(26A+X)	+		
150.	*S. arctica* (Zetterstedt, 1840)	25(24A+X)	+	Russia	Perepelov 2003
151.	*S. borisi* Marinov, 2001	20(18A+XY)	–	Bulgaria	Grozeva and Marinov 2007
152.	*S. flavomaculata* (Van der Linden, 1825)	25(24A+X)	–	Former USSR	Makalowskaja 1940
		– » –	–	Russia	Perepelov 2003
		– » –	+	Russia	Kuznetsova et al. 2020b
153.	*S. graeseri* Selys, 1887	25(24A+X)	–	Russia	Perepelov et al. 2001
154.	*S. meridionalis* Nielsen, 1935	25(24A+X)	–	Slovenia	Kiauta and Kiauta 1995
		– » –	–	Bulgaria	Grozeva and Marinov 2007
155.	*S. metallica* (Van der Linden, 1825)	26(24A+XX)*	–	Finland	Oksala 1945
		25(24A+X)	–	Finland	Nokkala et al. 2002
		– » –	–	Finland	Grozeva and Marinov 2007
		– » –	–	Russia	Perepelov and Bugrov 2001b
156.	*S. semicircularis* (Selys, 1871)	25(24A+X)	–	USA	Cruden 1968
157.	*S. uchidai* Fürster, 1909	25(24A+X)	+	Japan	Oguma 1915, 1930
		– » –	+	Japan	Kichijo 1942b
158.	*S. viridiaenea* (Uhler, 1858)	25(24A+X)	–	Japan	Oguma 1915, 1930
		– » –	–	Japan	Kichijo 1942b
** Libellulidae **					
159.	***Acisoma****panorpoides* Rambur, 1842	25(24A+X)	+	Bangladesh, India	Dasgupta 1957 (*A. p. panorpoides* Rambur, 1842)
		– » –	+	Nepal	Kiauta 1975 (*A. p. panorpoides*)
		– » –	+	Thailand	Kiauta and Kiauta 1983 (*A. p. panorpoides*)
		– » –	+	India	Tyagi 1982
160.	***Aethriamanta****brevipennis* (Rambur, 1842)	25(24A+X)	+	India	Dasgupta 1957
161.	***Anatya****guttata* (Erichson, 1848)	25(24A+X)	–	Surinam	Kiauta 1979a
162.	***Atoconeura****biordinata* Karsch, 1899	21(20A+X)	+	Sudan	Wasscher 1985
163.	***Brachydiplax****chalybea* Brauer, 1868	25(24A+X)	+	India	Dasgupta 1957
		– » –	+	India	Taygi 1982
		– » –	+	Thailand	Kiauta and Kiauta 1983
		– » –	+	India	Prasad and Thomas 1992
164.	*B. farinosa* Krueger, 1902	25(24A+X)	+	India	Dasgupta 1957
		– » –	+	India	Taygi 1982
		– » –	–	Thailand	Kiauta and Kiauta 1983
165.	*B. sobrina* (Rambur, 1842)	25(24A+X)	+	India	Ray Chaudhuri and Dasgupta 1949
		– » –	+	India	Taygi 1982
		– » –	+	Nepal	Kiauta and Kiauta 1982
166.	***Brachvmesia****furcata* (Hagen, 1861)	25(24A+X)	+	Surinam	Kiauta 1979a
		– » –	+	Argentina	Agopian and Mola 1988
		– » –	–	Brazil	Ferreira et al. 1979
		– » –	–	Brazil	Souza Bueno 1982
167.	*B. gravida* (Calvert, 1890)	25(24A+X)	+	USA	Cruden 1968 as *Cannacria gravida* (Calvert, 1890)
168.	*B. herbida* (Gundlach, 1889)	25(24A+X)	+	Jamaica	Cumming 1964 as *Cannacria herbida* (Gundlach, 1889)
169.	***Brachythemis****contaminata* (Fabricius, 1793)	25(24A+X)	+	India	Asana and Makino 1935
		– » –	+	India	Makino 1935
		– » –	+	India	Kichijo 1942b
		– » –	+	India	Dasgupta 1957
		– » –	+	Nepal	Kiauta 1975
		– » –	+	India	Tyagi 1982
		– » –	+	Thailand	Kiauta and Kiauta 1983
170.	*B. lacustris* (Kirby, 1899)	25(24A+X)	+	Sudan	Wasscher 1985
171.	***Bradinopyga****cornuta* Ris, 1911	25(24A+X)	+	Republic of South Africa	Boyes et al. 1980
172.	*B. geminata* (Rambur, 1842)	25(24A+X)	+	India	Dasgupta 1957
		– » –	+	India	Tyagi 1982
173.	***Brechmorhoga****mendax* (Hagen, 1861)	25(24A+X)	+	USA	Cruden 1968
		– » –	–		
174.	*B. nubecula* (Rambur, 1842)	25(24A+X)	+	Bolivia	Cumming 1964
175.	*B. pertinax* (Hagen, 1861)	25(24A+X)	–	Bolivia	Cumming 1964 (*B. p. peruviana* Ris, 1913)
176.	***Cannaphila****vibex* (Hagen, 1861)	25(24A+X)	+	Bolivia	Cumming 1964
177.	***Celithemis****amanda* (Hagen, 1861)	25(24A+X)	+	USA	Kiauta and Brink 1978
178.	*C. elisa* (Hagen, 1861)	25(24A+X)	+	USA	Cruden 1968
179.	*C. fasciata* Kirby, 1889	25(24A+X)	+	USA	Cruden 1968
180.	*C. ornata* (Rambur, 1842)	25(24A+X)	+	USA	Kiauta and Brink 1978
181.	***Crocothemis****erythraea* (Brulle, 1832)	25(24A+X)	+	India	Dasgupta 1957
		– » –	+	Kenya	Kiauta 1969b
		– » –	+	Italy	Kiauta 1971a
		– » –	+	India	Prasad and Thomas 1992
		– » –	+	Republic of South Africa	Boyes et al. 1980
		– » –	+	India	Tyagi 1982
182.	*C. sanguinolenta* (Burmeister, 1839)	25(24A+X)	+	Kingdom of Eswatini (Former Swaziland)	Boyes et al. 1980
183.	*C. servilia* (Drury, 1773)	25(24A+X)	+	India	Asana and Makino 1935
		– » –	+	India	Makino 1935
		– » –	+	India	Kichijo 1942b
		– » –	+	India	Ray Chaudhuri and Dasgupta 1949
		– » –	+	Nepal	Kiauta 1975
		– » –	+	Philippines	Kiauta and Kiauta 1980b
		– » –	+	Nepal	Kiauta and Kiauta 1982
		– » –	+	India	Tyagi 1982
		– » –	+	Thailand	Kiauta and Kiauta 1983
		– » –	+	Japan	Katatani 1987
		– » –	+	Japan	Higashi and Kayano 1993
		– » –	+	Japan, Taiwan	Higashi et al. 2001
		24(22A+neo-XY)	+	Japan	Omura 1955 (*C. s. mariannae* Kiauta, 983)
		– » –	–	Japan	Kiauta 1983 (*C. s. mariannae*)
		– » –	–	Japan	Katatani 1987 (*C. s. mariannae*)
		– » –	–	Japan	Higashi et al. 2001 (*C. s. mariannae*)
184.	***Dasythemis****esmeralda* Ris, 1910	25(24A+X)	+	Bolivia	Cumming 1964
185.	*D. mincki* (Karsch, 1890)	25(24A+X)	+	Brazil	Souza Bueno 1982
186.	*D. venosa* (Burmeister, 1839)	25(24A+X)	+	Brazil	Kiauta and Boyes 1972
187.	***Diastatops****intensa* Montgomery, 1940	25(24A+X)	+	Bolivia	Cumming 1964
188.	*D. obscura* (Fabricius, 1775)	25(24A+X)	+	Bolivia	Cumming 1964
189.	*D. pullata* (Burmeister, 1839)	23(22A+X)	+	Surinam	Kiauta 1979a
190.	***Diplacodes****bipunctata* (Brauer, 1865)	25(24A+X)	+	Australia	Kiauta 1969b
		29(28A+X)	+		
191.	*D. haematodes* (Burmeister, 1839)	25(24A+X)	+	Australia	Kiauta 1969b
		23(22A+X)	–		
192.	*D. lefebvrei* (Rambur, 1842)	25(24A+X)	+	Madagascar	Kiauta 1968c, 1969b
193.	*D. nebulosa* (Fabricius, 1793)	25(24A+X)	+	India	Dasgupta 1957
		– » –	+	India	Kiauta and Kiauta 1982
		– » –	+	India	Tyagi 1982
194.	*D. trivialis* (Rambur, 1842)	25(24A+X)	+	India	Asana and Makino 1935
		– » –	+	India	Makino 1935
		– » –	+	India	Dasgupta 1957
		– » –	+	Australia	Kiauta 1969c
		– » –	+	Nepal	Kiauta 1975
		– » –	+	India	Tyagi 1982
195.	***Dythemis****fugax* Hagen, 1861	25(24A+X)	+	USA	Cruden 1968
196.	*D. multipunctata* Kirby, 1894	25(24A+X)	+	Surinam	Kiauta 1979a
		– » –	+	Brazil	Ferreira et al. 1979
197.	*D. rufinefris* (Burmeister, 1839)	25(24A+X)	+	Jamaica	Cumming 1964
198.	*D. velox* Hagen, 1861	25(24A+X)	+	Bolivia	Cumming 1964
		– » –	+	Peru	Kiauta and Boyes 1972
199.	***Elasmothemis****cannacrioides* (Calvert, 1906)	21(20A+X)	–	Bolivia	Cumming 1964 as *Dythemis cannacrioides* Calvert, 1906
		23(22A+X)	+	Surinam	Kiauta 1979a as *D. cannacrioides*
		– » –	+	Brazil	Ferreira et al. 1979
200.	*E. williamsoni* (Ris, 1919)	22(20A+neo-XY)	–	Surinam	Kiauta 1979a as *Dythemis williamsoni* (Ris, 1919)
		25(24A+X)	–		
201.	***Erythemis****attala* (Selys, 1857)	25(24A+X)	–	Bolivia	Cumming 1964
		– » –	+	Argentina	Agopian and Mola 1988
202.	*E. collocata* (Hagen, 1861)	25(24A+X)	+	USA	Cruden 1968
203.	*E. credula* (Hagen, 1861)	25(24A+X)	+	Surinam	Kiauta 1979a
204.	*E. haematogastra* (Burmeister, 1839)	25(24A+X)	–	Surinam	Kiauta 1979a
205.	*E. peruviana* (Rambur, 1842)	25(24A+X)	–	Surinam	Kiauta 1979a
206.	*E. plebeja* (Burmeister, 1839)	25(24A+X)	–	Bolivia	Cumming 1964
207.	*E. simplicicollis* (Say, 1839)	25(24A+X)	+	USA	Cruden 1968
208.	*E. vesiculosa* (Fabricius, 1775)	25(24A+X)	–	Bolivia	Cumming 1964 as *Lepthemis vesiculosa* (Fabricius, 1775)
		– » –	–	Surinam	Kiauta 1979a as *L. vesiculosa*
		– » –	+	Brasil	Ferreira et al. 1979 as *L. vesiculosa*
209.	***Erythrodiplax****anomala* (Brauer, 1865)	25(24A+X)	+	Brazil	Souza Bueno 1982
210.	*E. atroterminala* Ris, 1911	25(24A+X)	+	Uruguay	Goni and Abenante 1982
		– » –	+	Argentina	Mola 1996
211.	*E. attenuata* (Kirby, 1889)	25(24A+X)	+	Surinam	Kiauta 1979a
		– » –	+	Brasil	Ferreira et al. 1979
212.	*E. basalis* (Kirby, 1897)	25(24A+X)	–	Bolivia	Cumming 1964
		– » –	+	Surinam	Kiauta 1979a (*E. b. basalis* (Kirby, 1897))
		– » –	+	Brasil	Ferreira et al. 1979 (*E. b. basalis*)
213.	*E. berenice* (Drury, 1770)	25(24A+X)	–	USA	Cruden 1968
		27(26A+X)	+	USA	Hung 1971
		25(24A+X)	+		
214.	*E. castanea* (Burmeister, 1839)	25(24A+X)	–	Bolivia	Cumming 1964
215.	*E. chromoptera* Borror, 1942	23(22A+X)	+	Uruguay	Goni and Abenante 1982
216.	*E. cleopatra* Ris, 1911	25(24A+X)	+	Peru	Kiauta and Boyes 1972
217.	*E. connata* (Burmeister, 1839)	25(24A+X)	+	Chile	Kiauta and Boyes 1972 (*E. c. connata* (Burmeister, 1839))
		– » –	+	USA	Kiauta and Brink 1978 (*E. c. minuscula* (Rambur, 1842))
218.	*E. coralline* (Brauer, 1865)	25(24A+X)	+	Argentina	Mola 1996
219.	*E. famula* (Erichson, 1848)	25(24A+X)	+	Brazil	Souza Bueno 1982
220.	*E. fusca* (Rambur, 1842)	25(24A+X)	–	Bolivia	Cumming 1964 as *E. connata fusca* (Rambur, 1842)
		– » –	–	Guatemala	Cruden 1968 as *E. c. fusca*
		– » –	+	Surinam	Kiauta 1979a as *E. c. fusca*
		– » –	+	Brazil	Ferreira et al. 1979 as *E. c. fusca*
		– » –	+	Brazil	Souza Bueno 1982
		– » –	+	Argentina	Mola 1996
221.	*E. fervida* (Erichson, 1848)	25(24A+X)	+	Jamaica	Cumming 1964
222.	*E. justiniana* (Selys, 1857)	25(24A+X)	+	Jamaica	Cumming 1964
223.	*E. juliana* Ris, 1911	25(24A+X)	+	Brazil	Souza Bueno 1982
224.	*E. latimaculata* Ris, 1911	25(24A+X)	+	Surinam	Kiauta 1979a
		– » –	+	Brasil	Ferreira et al. 1979
225.	*E. lygaea* Ris, 1911	25(24A+X)	+	Argentina	Capitulo et al. 1991
		– » –	+	Argentina	Mola 1996
226.	*E. media* Borror, 1942	21(20A+X)	+	Bolivia	Cumming 1964
		22(20A+XX)*	+	Brazil	Kiauta and Boyes 1972
		21(20A+X)	+	Surinam	Kiauta 1979a
		– » –	+	Brasil	Ferreira et al. 1979
		22(20A+neo-XY)	+	Argentina	Mola 1996
227.	*E. melanorubra* Borror, 1942	25(24A+X)	+	Bolivia	Cumming 1964
		– » –	+	Venezuela	Kiauta and Boyes 1972
		– » –	+	Argentina	Capitulo et al. 1991
		– » –	+	Argentina	Mola 1996
228.	*E. minuscula* (Rambur, 1842)	25(24A+X)	+	USA	Kiauta and Brink 1978
		22(20A+neo-XY)	+	Argentina	Mola and Agopian 1985
229.	*E. nigricans* (Rambur, 1842)	25(24A+X)	+	Uruguay	Goni and Abenante 1982
229.	*E. nigricans* (Rambur, 1842)	– » –	+	Argentina	Mola 1996
		– » –	–	Argentina	De Gennaro 2004
		– » –	+	Argentina	De Gennaro et al. 2008
230.	*E. ochracea* (Burmeister, 1839)	25(24A+X)	+	Argentina	Mola 1996
231.	*E. paraguayensis* (Foerster, 1904)	23(22A+X)	+	Bolivia	Cumming 1964
		– » –	+	Surinam	Kiauta 1979a
232.	*E. umbrata* (Linnaeus, 1758)	25(24A+X)	+	Bolivia	Cumming 1964
		– » –	+	Dominica	Cruden 1968
		– » –	+	Surinam	Kiauta 1979a
		– » –	+	Brazil	Ferreira et al 1979
		– » –	+	Argentina	Mola 1996
233.	*E. unimaculata* (DeGeer, 1773)	25(24A+X)	+	Bolivia	Cumming 1964
		– » –	+	Surinam	Kiauta 1979a
234.	***Hydrobasileus****croceus* (Brauer, 1867)	25(24A+X)	+	India	Prasad and Thomas 1992
235.	***Ladona****julia* (Uhler, 1857)	25(24A+X)	+	USA	Cruden 1968
236.	***Lathrecista****asiatica* (Fabricius, 1798)	25(24A+X)	+	India	Dasgupta 1957
		– » –	+	India	Tyagi 1982
237.	***Leucorrhinia****albifrons* (Burmeister, 1839)	25(24A+X)	+	Former USSR	Makalowskaja 1940
238.	*L. dubia* (Van der Linden, 1825)	26(24A+XX)*	–	Finland	Oksala 1939a, 1945
		25(24A+X)	+	Russia	Kuznetsova et al. 2020b
239.	*L. frigida* Hagen, 1890	21(20A+X)	–	USA	Cruden 1968
		23(22A+X)	+		
240.	*L. glacialis* Hagen, 1890	25(24A+X)	+	USA	Cruden 1968
241.	*L. hudsonica* (Selys, 1850)	25(24A+X)	+	USA	Cruden 1968
		– » –	–		
242.	*L. intacta* (Hagen, 1861)	25(24A+X)	+	USA	Cruden 1968
		– » –	–		
243.	*L. pectoralis* (Charpentier, 1825)	26(24A+XX)*	–	Finland	Oksala 1945
244.	*L. proxima* Calvert, 1890	25(24A+X)	+	USA	Cruden 1968
245.	*L. rubicunda* (Linnaeus, 1857)	25(24A+X)	–	Finland	Oksala 1939a
		– » –	–	Former USSR	Makalowskaja 1940
		– » –	–	Russia	Kuznetsova et al. 2018
246.	***Libellula****angelina* Selys, 1883	25(24A+X)	+	Japan	Oguma 1915, 1930
		– » –	+	Japan	Kichijo 1942a
247.	*L. auripennis* Burmeister, 1839	25(24A+X)	+	USA	Kiauta and Brink 1978
248.	*L. axilena* Westwood, 1837	23(22A+X)	–	USA	Cumming 1964
249.	*L. basalis* (Say, 1840)	25(24A+X)	–	USA	Smith 1916
250.	*L. composita* (Hagen, 1873)	25(24A+X)	+	USA	Cruden 1968
251.	*L. croceipennis* Selys, 1868	25(24A+X)	+	USA	Cruden 1968
252.	*L. cyanea* Fabricius, 1775	25(24A+X)	–	USA	Cruden 1968
253.	*L. depressa* Linnaeus, 1758	23(22A+X)	–	Belgium	Carnoy 1885
		– » –	–	England	Hogben 1921
		25(24A+X)	+	Austria	Kiauta 1968c, 1969b
		23(22A+X)	–		
		25(24A+X)	+	France	Kiauta 1973b
		– » –	+	Croatia	Francovič and Jurečic 1986, 1989
		– » –	+	Russia	Perepelov et al. 1998
		– » –	+	Russia	Kuznetsova et al. 2018
254.	*L. flavida* Rambur, 1842	25(24A+X)	+	USA	Cruden 1968
255.	*L. forensis* Hagen, 1861	25(24A+X)	+	USA	Cruden 1968
256.	*L. fulva* Muller, 1764	25(24A+X)	+	Switzerland	Kiauta and Kiauta 1979
		27(26A+X)	+	Croatia	Francovič and Jurečic 1986, 1989
257.	*L. insecta* Hagen, 1861	25(24A+X)	–	USA	Cumming 1964
		– » –	–	USA	Cruden 1968
258.	*L. luctuosa* Burmeister, 1839	25(24A+X)	–	USA	Smith 1916
259.	*L. pulchella* Drury, 1773	25(24A+X)	+	USA	Cruden 1968
		– » –	+	Canada	Kiauta 1969a
260.	*L. quadrimaculata* Linnaeus, 1758	25(24A+X)	+	Japan	Oguma 1915, 1930 (*L. q. asahinai* Schmidt, 1957)
260.	*L. quadrimaculata* Linnaeus, 1758	25(24A+X)	+	Japan	Kichijo 1942d (*L. q. asahinai*)
		– » –	+	Japan	Omura 1955 (*L. q. asahinai*)
		– » –	+	Japan	Kiauta 1968b, c (*L. q. asahinai*)
		– » –	+	Former USSR	Fuchsówna and Sawczyńska 1928 (*L. q. quadrimaculata* Linnaeus, 1758)
		– » –	+	Finland	Oksala 1939a, b, 1945 (*L. q. quadrimaculata*)
		– » –	+	Former USSR	Makalowskaja 1940 (*L. q. quadrimaculata*)
		– » –	+	Netherlands	Kiauta 1968b, c (*L. q. quadrimaculata*)
		– » –	+	USA	Cruden 1968 (*L. q. quadrimaculata*)
		– » –	+	Russia	Perepelov et al. 1998 (*L. q. quadrimaculata*)
		– » –	+	Russia	Kuznetsova et al. 2018 (*L. q. quadrimaculata*)
261.	*L. saturata* Uhler, 1857	25(24A+X)	+	USA	Cruden 1968
262.	*L. semifasciata* Burmeister, 1839	25(24A+X)	+	USA	Cruden 1968
263.	*L. vibrans* Fabricius, 1793	25(24A+X)	+	USA	Cruden 1968
264.	***Lyriothemis****pachygastra* (Selys, 1878)	25(24A+X)	–	Japan	Omura 1955
265.	***Macrothemis****declivata* Calvert, 1909	23(22A+X)	+	Brazil	Kiauta and Boyes 1972
266.	*M. hemichlora* (Burmeister, 1839)	6(4A+neo-XY)	–	Bolivia	Cumming 1964
267.	*M. imitans* Karsch, 1890	25(24A+X)	+	Brazil	Kiauta and Boyes 1972 (*M. i. imitans* Karsch, 1890)
268.	*M. mortoni* Ris, 1913	25(24A+X)	+	Bolivia	Cumming 1964
269.	*M. musiva* Calvert, 1898	25(24A+X)	+	Bolivia	Cumming 1964
270.	*Macrothemis* sp.	25(24A+X)	+	Argentina	Mola 2007
271.	***Miathyria****artemis* (Selys, 1857)	25(24A+X)	+	Surinam	Kiauta 1979a
272.	*M. marcella* (Selys, 1857)	25(24A+X)	+	Bolivia	Cumming 1964
		– » –	+	Surinam	Kiauta 1979a
		– » –	+	Argentina	Mola and Agopian 1985
		– » –	+	Brazil	Ferreira et al. 1979
273.	***Micrathyria****artemis* Ris, 1911	25(24A+X)	+	Brazil	Ferreira et al. 1979
		– » –	+	Brazil	Souza Bueno 1982
274.	*M. atra* (Martin, 1897)	25(24A+X)	+	Bolivia	Cumming 1964
275.	*M. catenata* Calvert, 1909	25(24A+X)	+	Brazil	Souza Bueno 1982
		– » –	+	Argentina	Mola 2007
276.	*M. didyma* (Selys, 1857)	25(24A+X)	+	Jamaica	Cumming 1964
277.	*M. exima* Kirby, 1897	25(24A+X)	+	Surinam	Kiauta 1979a
278.	*M. hagenii* Kirby, 1890	25(24A+X)	+	Jamaica	Cumming 1964
279.	*M. hesperis* Ris, 1911	25(24A+X)	+	Surinam	Kiauta 1979a
		– » –	+	Brazil	Ferreira et al. 1979
		– » –	+	Argentina	Mola et al. 1999
280.	*M. hypodydima* Calvert 1906	23(22A+X)	+	Brazil	Souza Bueno 1982
		25(24A+X)	+	Argentina	Agopian and Mola 1988
281.	*M. iheringi* Santos, 1946	23(22A+X)	+	Bolivia	Cumming 1964
282.	*M. laevigata* Calvert, 1909	25(24A+X)	+	Bolivia	Cumming 1964
		– » –	+	Brazil	Kiauta and Boyes 1972
283.	*M. longifasciata* Calvert, 1909	24(22A+neo-XY)	–	Argentina	Agopian and Mola 1988
284.	*M. ocellata* (Martin, 1897)	25(24A+X)	+	Bolivia	Cumming 1964 (*M. o. dentiens* Calvert, 1909)
285.	*M. spuria* (Selys, 1900)	25(24A+X)	+	Bolivia	Cumming 1964
		– » –	+	Argentina	Mola et al. 1999
286.	*M. stawiarskii* Santos, 1953	25(24A+X)	+	Brazil	Souza Bueno 1982
287.	*M. ungulata* Foerster, 1907	23(20A+X_1_X_2_Y)	–	Argentina	Mola et al. 1999
288.	M. cf. eximia Kirby, 1879	21(20A+X)	–	Bolivia	Cumming 1964
289.	*M.* sp. (*ungulata* Foerster, 1907-group)	23(22A+X)	–	Bolivia	Cumming 1964
290.	***Nannothemis****bella* (Uhler, 1857)	25(24A+X)	+	USA	Cruden 1968
291.	***Nesciothemis****farinosa* (Foerster, 1898)	25(24A+X)	+	Kenya	Kiauta 1969c
		– » –	+	Kenya	Wasscher 1985
292.	***Nesogonia****blackburni* (McLachlan, 1883)	25(24A+X)	+	Hawaii	Kiauta 1969d
293.	***Neurothemis****fulvia* (Drury, 1773)	25(24A+X)	+	Nepal	Kiauta 1974, 1975
294.	*N. intermedia* (Rambur, 1842)	25(24A+X)	+	Nepal	Kiauta 1974, 1975 (*N. i. intermedia* (Rambur, 1842))
		– » –	+	Nepal	Kiauta and Kiauta 1982 (*N. i. degener* (Sel, 1842))
295.	*N. terminata* Ris, 1911	25(24A+X)	+	Philippines	Kiauta and Kiauta 1980b
296.	*N. tullia* (Drury, 1773)	28(26A+neo-XY)	+	India	Ray Chaudhuri and Dasgupta 1949
		– » –	+	India	Kiauta 1969a (*N. t. tullia* (Drury, 1773))
		– » –	+	India	Tyagi 1982 (*N. t. tullia*)
		25(24A+X)	+	Thailand	Kiauta and Kiauta 1983
297.	***Oligoclada****amphinome* Ris, 1919	25(24A+X)	+	Surinam	Kiauta 1979a
298.	*O. laetitia* Ris, 1911	23(22A+X)	+	Argentina	Mola and Agopian 1985
		21(20A+X)	–	Brazil	Souza Bueno 1982
299.	*O. monosticha* Borror, 1931	23(22A+X)	+	Surinam	Kiauta 1979a
		– » –	+	Brazil	Ferreira et al. 1979
300.	*O. pachystigma* Karsch, 1890	23(22A+X)	+	Brazil	Souza Bueno 1982
301.	***Orthemis****aequilibris* Calvert, 1909	12(10A+neo-XY)	–	Surinam	Kiauta 1979a
302.	*O. ambinigra* Calvert, 1909	12(10A+neo-XY)	–	Argentina	Agopian and Mola 1984
303.	*O. biolleyi* Calvert, 1906	23(22A+X)	+	Bolivia	Cumming 1964
304.	*O. cultiformis* Calvert, 1906	23(22A+X)	+	Bolivia	Cumming 1964
		– » –	+	Surinam	Kiauta 1979a
		– » –	+	Brazil	Ferreira et al. 1979
305.	*O. discolor* Burmeister, 1839	23(22A+X)	+	Argentina	Mola 2007
306.	*O. ferruginea* (Fabricius, 1775)	10(8A+neo-XY)***	–	Bolivia	Cumming 1964
		23(22A+X)	–	USA	
		– » –	+	Guatemala, Dominica	Cruden 1968
		– » –	+	Peru	Kiauta 1969a, 1971c
		– » –	+	Peru	Kiauta and Boyes 1972
		23(22A+X)	+	Surinam	Kiauta 1979a
		25(24A+X)	+		
		23(22A+X)	+	Brazil	Ferreira et al. 1979
		23(22A+X)	–	Brazil, Argentina	Mola and Agopian 1985
		24(22A+XX)*	+		
307.	*O. levis* Calvert, 1906	6(4A+neo-XY)***	–	Bolivia	Cumming 1964
		8(6A+neo-XY)***	–		
308.	*O. nodiplaga* Karsch, 1891	41(40A+X)	–	Argentina	Agopian and Mola 1984
309.	***Orthetrum****abbotti* Calvert, 1892	25(24A+X)	+	Kingdom of Eswatini (Former Swaziland)	Boyes et al. 1980
310.	*O. albistylum* (Selys, 1848)	25(24A+X)	+	Italy	Kiauta 1971a (*O. a. albistylum* (Selys, 1848))
		– » –	+	Russia	Perepelov et al. 1998
		– » –	+	Japan	Oguma 1915, 1917, 1930 (*O. a. speciosum* (Uhler, 1858))
		– » –	+	India	Kichijo 1942b (*O. a. speciosum*)
		– » –	+	Japan	Omura 1955 (*O. a. speciosum*)
311.	*O. azureum* (Rambur, 1842)	25(24A+X)	+	Madagascar	Kiauta 1969b, c
312.	*O. brachiale* (Beauvois, 1805)	21(20A+X)	–	Kenya	Kiauta 1969b, c
		25(24A+X)	+	Burkina Faso (Former Voltiac Republic)	Kiauta and Ochssée 1979 (*O. b. brachiale* (Beauvois, 1805))
313.	*O. brunneum* (Fonscolombe, 1837)	25(24A+X)	+	Italy	Kiauta 1971a
		– » –	+	Russia	Perepelov et al. 1998
314.	*O. cancellatum* (Linnaeus, 1758)	25(24A+X)	+	Finland	Oksala 1939a
		– » –	+	India	Dasgupta 1957
		– » –	+	Netherlands	Kiauta 1969a, b
		– » –	+	India	Tyagi 1982
		– » –	+	Russia	Kuznetsova et al. 2018
315.	*O. chrysostigma* (Burmeister, 1839)	25(24A+X)	+	Burkina Faso (Former Voltiac Republic)	Kiauta and Ochssée 1979
		– » –	+	Kingdom of Eswatini (Former Swaziland)	Boyes et al. 1980
		– » –	+	Kenya	Wasscher 1985
316.	*O. coerulescens* (Fabricius, 1798)	25(24A+X)	+	Austria	Kiauta 1969c
		23(22A+X)	–		
		25(24A+X)	+	Italy	Kiauta 1971a
		27(26A+X)	+		
317.	*O. glaucum* (Brauer, 1865)	25(24A+X)	+	India	Dasgupta 1957
		– » –	+	India	Tyagi 1978a, b
		– » –	+	India	Handa and Batra 1980
		– » –	+	India	Tyagi 1982
		– » –	+	India	Handa et al. 1984
		– » –	+	India	Walia and Sandhu 2002
		– » –	+	India	Kumari and Gautam 2017
318.	*O. guineese* (Ris, 1909)	25(24A+X)	+	Burkina Faso (Former Voltiac Republic)	Kiauta and Ochssée 1979
319.	*O. japonicum* (Uhler, 1858)	25(24A+X)	+	Japan	Oguma 1917, 1930 (*O. j. internum* McLachlan, 1894)
		– » –	+	Japan	Kichijo 1942b (*O. j. internum*)
		– » –	+	Japan	Omura 1955 (*O. j. internum*)
		– » –	+	Nepal	Kiauta 1975 (*O. j. internum*)
		– » –	+	Nepal	Kiauta and Kiauta 1976 (*O. j. internum*)
320.	*O. julia* Kirby, 1900	25(24A+X)	+	Kingdom of Eswatini (Former Swaziland)	Boyes et al. 1980 (*O. j. falsum* (Longfeild, 1955))
		– » –	+	Kenya	Wasscher 1985 (*O. j. falsum*)
321.	*O. luzonicum* (Brauer, 1868)	25(24A+X)	+	Nepal	Kiauta 1975
		– » –	+	Nepal	Kiauta and Kiauta 1982
		– » –	+	India	Thomas and Prasad 1981
		– » –	+	India	Prasad and Thomas 1992
322.	*O. melania* (Selys, 1883)	25(24A+X)	+	Japan	Oguma 1917
		– » –	+	Japan	Omura 1955
		– » –	+	Russia	Perepelov 2003
323.	*O. monardi* (Schmidt, 1951)	25(24A+X)	+	Burkina Faso (Former Voltiac Republic)	Kiauta and Ochssée 1979
324.	*O. poecilops* (Ris, 1916)	25(24A+X)	+	Japan	Suzuki et al. 1991 (*O. p. miyajimaensis* Yuki et Doi, 1938)
325.	*O. pruinosum* (Burmeister, 1839)	25(24A+X)	+	India	Dasgupta 1957 (*O. p. neglectum* (Rambur, 1842))
		– » –	+	Taiwan	Kiauta 1969a, c (*O. p. neglectum*)
		– » –	+	India	Tyagi 1982 (*O. p. neglectum*)
		– » –	+	India	Prasad and Thomas 1992 (*O. p. neglectum*)
		– » –	+	India	Tyagi 1978a, b (*O. p. neglectum*)
		– » –	+	Nepal	Kiauta and Kiauta 1982 (*O. p. neglectum*)
		– » –	+	India	Walia and Sandhu 2002 (*O. p. neglectum*)
		– » –	+	India	Kumari and Gautam 2017 (*O. p. neglectum*)
326.	*O. sabina* (Drury, 1773)	25(24A+X)	+	India	Asana and Makino 1935
		– » –	+	India	Makino 1935
		– » –	+	India	Kichijo 1942b
		– » –	+	India	Ray Chaudhuri and Dasgupta 1949
		– » –	+	Nepal	Kiauta 1975
326.	*O. sabina* (Drury, 1773)	– » –	+	India	Tyagi 1982
		– » –	+	India	Prasad and Thomas 1992
		– » –	+	India	Walia and Sandhu 2002 (*O. s. sabina* (Drury, 1773))
327.	*O. taeniolatum* (Schneider, 1845)	25(24A+X)	+	Greece	Kiauta 1972a
		– » –	+	Nepal	Kiauta 1975
		– » –	+	India	Tyagi 1978a, b
		– » –	+	India	Handa and Batra 1980
		– » –	+	India	Tyagi 1982
		– » –	+	India	Handa et al. 1984
		– » –	+	India	Thomas and Prasad 1986
		– » –	+	India	Walia and Sandhu 2002a
		– » –	+	India	Walia et al. 2015
328.	*O. testaceum* (Burmeister, 1839)	25(24A+X)	+	Nepal	Kiauta and Kiauta 1982
329.	*O. triangulare* (Selys, 1878)	25(24A+X)	+	Japan	Omura 1955 (*O. t. melania* (Selys, 1883))
		– » –	+	Taiwan	Kiauta 1969a, b (*O. t. triangulare* (Selys, 1878))
		– » –	+	Nepal	Kiauta 1975 (*O. t. triangulare*)
		– » –	+	India	Tyagi 1978a, b (*O. t. triangulare*)
		– » –	+	India	Handa and Batra 1980 (*O. t. triangulare*)
		– » –	+	India	Tyagi 1982 (*O. t. triangulare*)
		– » –	+	India	Walia and Sandhu 2002 (*O. t. triangulare*)
330.	***Pachydiplax****longipennis* (Burmeister, 1839)	25(24A+X)	–	USA	Cumming 1964
		– » –	+	USA	Cruden 1968
		– » –	+	USA	Kiauta and Brink 1978
331.	***Palpopleura****jucunda* Rambur, 1842	25(24A+X)	+	Kingdom of Eswatini (Former Swaziland)	Boyes et al. 1980
332.	*P. lucia* (Drury, 1773)	25(24A+X)	+	Burkina Faso (Former Voltiac Republic)	Kiauta and Ochssée 1979 (*P. l. portia* (Drury, 1773))
		– » –	+	Kenya	Wasscher 1985 (*P. l. portia*)
333.	*P. sexmaculata* (Fabricius, 1787)	25(24A+X)	+	Nepal	Kiauta 1974, 1975
		– » –	+	India	Tyagi 1982 (*P. s. sexmaculata* (Fabricius, 1787))
334.	***Pantala****flavescens* (Fabricius, 1798)	25(24A+X)	+	India	Asana and Makino 1935
		– » –	+	India	Makino 1935
		– » –	+	India	Kichijo 1942b
		– » –	+	India	Dasgupta 1957
		– » –	+	India	Seshachar and Bagga 1963
		– » –	+	Bolivia	Cumming 1964
		– » –	+	Madagascar	Kiauta 1969b
		– » –	+	Surinam	Kiauta 1979a
		– » –	+	Brazil	Ferreira et al. 1979
		– » –	+	Kingdom of Eswatini (Former Swaziland)	Boyes et al. 1980
		– » –	+	Brazil	Souza Bueno 1982
		– » –	+	Argentina	Agopian and Mola 1988
		– » –	+	India	Prasad and Thomas 1992
		– » –	+	Russia	Perepelov and Bugrov 2001b
		23(22A+X)	+	India	Walia et al. 2011
335.	*P. hymenaea* (Say, 1836)	25(24A+X)	+	Bolivia	Cumming 1964
		– » –	+	USA	Cruden 1968
336.	***Perithemis****cornelia* Ris, 1910	25(24A+X)	–	Bolivia	Cumming 1964
337.	*P. domitia* (Drury, 1773)	25(24A+X)	+	Jamaica	Cumming 1964
338.	*P. electra* Ris, 1928	25(24A+X)	–	Bolivia	Cumming 1964
339.	*P. icteroptera* (Selys in Sagra, 1857)	25(24A+X)	+	Argentina	Mola and Agopian 1985
340.	*P. lais* (Petry, 1834)	17(16A+X)	–	Bolivia	Cumming 1964
		– » –	–	Surinam	Kiauta 1979a
		– » –	–	Brazil	Ferreira et al. 1979
341.	*P. mooma* Kirby, 1889	25(24A+X)	+	Bolivia	Cumming 1964
		– » –	+	Surinam	Kiauta 1979a
		– » –	–	Brazil	Ferreira et al. 1979
		– » –	+	Argentina	Mola and Agopian 1985
342.	*P. tenera* (Say, 1839)	25(24A+X)	+	USA	Kiauta and Brink 1978
343.	*P. seminole* Calvert, 1907	25(24A+X)	+	USA	Cumming 1964
344.	*Perithemis* sp.	25(24A+X)	–	Bolivia	Cumming 1964
345.	***Planiplax****erythropyga* (Karsch, 1891)	25(24A+X)	+	Argentina	Mola et al. 1999
		– » –	+	– » –	De Gennaro 2004
346.	*P. sanguiniventris* (Calvert, 1907)	25(24A+X)	+	USA	Cruden 1968
347.	***Plathemis****lydia* (Drury, 1773)	25(24A+X)	+	USA	McGill 1907
		– » –	+	USA	Cruden 1968
348.	***Potamarcha****congener* (Rambur, 1842)	25(24A+X)	+	India	Asana and Makino 1935 as *P. obscura* (Rambur, 1842)
		– » –	+	India	Makino 1935 as *P. obscura*
		– » –	+	India	Kichijo 1942b as *P. obscura*
		– » –	+	India	Dasgupta 1957 as *P. obscura*
		– » –	+	India	Tyagi 1982 as *P. obscura*
		– » –	+	India	Prasad and Thomas 1992
		– » –	+	India	Sandhu and Walia 1995
349.	***Pseudothemis****zonata* (Burmeister, 1839)	24(22A+neo-XY)	–	Japan	Omura 1955
350.	***Pseudotramea****prateri* Fraser, 1920	25(24A+X)	+	Nepal	Kiauta 1974, 1975
351.	***Rhodopygia****cardinalis* (Erichson, 1848)	25(24A+X)	+	Bolivia	Cumming 1964
352.	*R. geijskesi* Belle, 1964	25(24A+X)	+	Surinam	Kiauta 1979a
353.	***Rhodothemis****rufa* (Rambur, 1842)	25(24A+X)	+	India	Prasad and Thomas 1992
354.	***Rhyothemis****fuliginosa* Selys, 1883	25(24A+X)	+	Japan	Toyoshima and Hirai 1953
		– » –	+	Japan	Omura 1955
		– » –	+	Japan	Hirai 1956
		25(24A+X)	+	Japan	Kiauta 1969c
		23(22A+X)	+		
355.	*R. variegata* (Linnaeus et Johansson, 1763)	25(24A+X)	+	India	Ray Chaudhuri and Dasgupta 1949
		– » –	+	Nepal	Kiauta 1975
356.	***Scapanea****frontalis* (Burmeister, 1839)	25(24A+X)	+	Jamaica	Cumming 1964
357.	***Sympetrum****commixtum* (Selys, 1884)	25(24A+X)	–	India	Tyagi 1978a, b, 1982
358.	*S. corruptum* (Hagen, 1861	25(24A+X)	+	USA	Cruden 1968 as *Tarnetrum corruptum* (Hagen, 1861)
		– » –	+	USA	Kiauta 1969a, c as *T. corruptum*
359.	*S. costiferum* (Hagen, 1861)	25(24A+X)	+	USA	Cruden 1968
360.	*S. croceolum* (Selys, 1840)	25(24A+X)	+	Russia	Perepelov 2003
361.	*S. danae* (Sulzer, 1776)	25(24A+X)	+	Former USSR	Makalowskaja 1940
		– » –	+	Finland	Oksala 1945
		– » –	+	USA	Cruden 1968
		– » –	+	Russia	Perepelov 2003
		– » –	+	Russia	Kuznetsova et al. 2018
362.	*S. eroticum* (Selys, 1883)	21(20A+X)	–	Japan	Kichijo 1942b, c
		– » –	–	Japan	Hirai 1956
		– » –	–	Japan	Kiauta 1969c
363.	*S. flaveolum* (Linnaeus, 1758)	25(24A+X)	+	Former USSR	Makalowskaja 1940
		– » –	+	Russia	Perepelov 2003
364.	*S. fonscolombii* (Selys, 1840)	25(24A+X)	+	Russia	Perepelov 2003
365.	*S. frequens* (Selys, 1883)	23(22A+X)	–	Japan	Oguma 1917, 1930
		– » –	–	Japan	Kichijo 1942a, b
		– » –	–	Japan	Kiauta 1969c
366.	*S. infuscatum* (Selys, 1883)	25(24A+X)	+	Russia	Perepelov 2003
367.	*S. internum* Montgomery, 1943	27(26A+X)	+	Canada	Kiauta 1973a
368.	*S. madidum* (Hagen, 1861)	25(24A+X)	+	USA	Cruden 1968
368.	*S. madidum* (Hagen, 1861)	– » –	+	Canada	Kiauta 1973a
369.	*S. meridionale* (Selys, 1841)	25(24A+X)	+	Switzerland	Kiauta 1966
370.	*S. obtrusum* (Hagen, 1867)	25(24A+X)	+	USA	Cruden 1968
371.	*S. parvulum* Bartenev, 1912	25(24A+X)	+	Japan	Kiauta 1968c
372.	*S. pedemontanum* Müller in Allioni, 1766	25(24A+X)	+	Japan	Oguma 1917, 1930 (*S. p. elatum* (Selys, 1872))
		– » –	+	Japan	Kichijo 1942b (*S. p. elatum*)
		– » –	+	Japan	Kiauta and Brink 1975 (*S. p. elatum*)
		– » –	+	Switzerland	Kiauta and Brink 1975 (*S. p. pedemontanum* (Müller, 1766))
		– » –	+	Russia	Perepelov et al. 1998 (*S. p. pedemontanum*)
		– » –	+	Russia	Perepelov and Bugrov 2001b
373.	*S. rubicundulum* (Say, 1839)	25(24A+X)	+	USA	Cruden 1968
374.	*S. sanguineum* (Müller, 1764)	25(24A+X)	+	Italy	Kiauta 1971a
		– » –	+	Russia	Perepelov and Bugrov 2001b
375.	*S. semicinctum* (Say, 1839)	25(24A+X)	+	USA	Smith 1916
		– » –	+	USA	Cruden 1968
376.	*S. striolatum* (Charpentier, 1840)	25(24A+X)	–	Luxembourg	Kiauta 1966
377.	*S. vicinum* (Hagen, 1861)	25(24A+X)	+	USA	Cruden 1968
378.	*S. vulgatum* (Linnaeus, 1758)	25(24A+X)	+	Netherland	Kiauta 1972c
		– » –	+	Russia	Perepelov 2003
		– » –	+	Russia	Kuznetsova et al. 2018
379.	***Tarnetrum****illotum* (Hagen, 1861)	25(24A+X)	+	Jamaica	Cumming 1964
		– » –	+	USA	Cruden 1968
380.	***Tauriphila****australis* (Hagen, 1867)	25(24A+X)	+	Bolivia	Cumming 1964
381.	*T. azteca* Calvert, 1906	25(24A+X)	+	Mexico	Cruden 1968
382.	*T. risi* Martin 1896	25(24A+X)	+	Argentina, Uruguay	Mola and Agopian 1985
383.	***Tholymis****citrina* Hagen, 1867	25(24A+X)	+	Surinam	Kiauta 1979a
		– » –	+	Brazil	Ferreira et al. 1979
384.	*Th. tillagra* (Fabricius, 1798)	25(24A+X)	+	India	Prasad and Thomas 1992
		– » –	+	Nepal	Kiauta and Kiauta 1982
		– » –	+	Thailand	Kiauta and Kiauta 1983
385.	***Tramea****abdominalis* (Rambur, 1842)	25(24A+X)	–	Bolivia	Cumming 1964
386.	*T. basilaris* (Palisot de Beauvois, 1817)	25(24A+X)	+	India	Das 1956 (*T. b. burmeisteri* (Kirby, 1889))
		– » –	+	India	Dasgupta 1957 (*T. b. burmeisteri*)
		– » –	+	Nepal	Kiauta and Kiauta 1982 (*T. b. burmeisteri*)
		– » –	+	India	Prasad and Thomas 1992 (*T. b. burmeisteri*)
387.	*T. binotata* (Rambur, 1842)	25(24A+X)	+	Surinam	Kiauta 1979a
		– » –	–	Brazil	Ferreira et al. 1979
388.	*T. carolina* (Linnaeus, 1763)	25(24A+X)	–	USA	Cumming 1964
		– » –	–	USA	Cruden 1968
389.	*T. cophysa* (Hagen, 1867)	25(24A+X)	+	Bolivia	Cumming 1964
390.	*T. lacerata* (Hagen, 1861)	25(24A+X)	–	USA	Cruden 1968
391.	*T. limbata* (Desjardins, 1832)	25(24A+X)	+	India	Asana and Makino 1935
		– » –	+	India	Makino 1935
		– » –	+	India	Kichijo 1942b
392.	*T. virginia* (Rambur, 1842)	25(24A+X)	+	India	Oguma and Asana 1932
		– » –	+	India	Kichijo 1942b
		– » –	+	India	Dasgupta 1957
393.	***Trithemis****annulata* (Palisot de Beauvois, 1805)	25(24A+X)	–	Republic of South Africa	Boyes et al. 1980
		– » –	+	Kenya	Wasscher 1985
394.	*T. arteriosa* (Burmeister, 1839)	25(24A+X)	+	Kingdom of Eswatini (Former Swaziland)	Boyes et al. 1980
395.	*T. atra* Pinhey, 1961	25(24A+X)	+	Burkina Faso (Former Voltiac Republic)	Kiauta and Ochssée 1979
396.	*T. aurora* (Burmeister, 1839)	25(24A+X)	+	India	Oguma and Asana 1932
		– » –	+	Nepal	Kiauta 1975
		– » –	+	India	Tyagi 1982
397.	*T. dorsalis* (Rambur, 1842)	25(24A+X)	+	Kingdom of Eswatini (Former Swaziland)	Boyes et al. 1980
398.	*T. festiva* (Rambur, 1842)	25(24A+X)	+	Nepal	Kiauta 1974, 1975
		– » –	+	India	Tyagi 1982
		– » –	+	India	Prasad and Thomas 1992
399.	*T. furva* Karsch, 1899	25(24A+X)	+	Sudan	Wasscher 1985
400.	*T. imiata* Pinhey, 1961	25(24A+X)	–	Burkina Faso (Former Voltiac Republic)	Kiauta and Ochssée 1979
401.	*T. kirbyi* Selys, 1891	25(24A+X)	–	Burkina Faso (Former Voltiac Republic)	Kiauta and Ochssée 1979 (*T. k. ardens* Gerstaecker, 1891)
		– » –	+	Kenya	Wasscher 1985 (*T. k. ardens*)
402.	*T. pallidinervis* (Kirby, 1889)	25(24A+X)	+	India	Asana and Makino 1935
		– » –	+	India	Makino 1935
		– » –	+	India	Kichijo 1942b
		– » –	+	India	Dasgupta 1957
		– » –	+	Philippines	Kiauta and Kiauta 1980b
403.	*T. werneri* Ris, 1912	25(24A+X)	+	Kenya	Wasscher 1985
404.	***Uracis****imbuta* (Burmeister, 1839)	25(24A+X)	+	Surinam	Kiauta 1979a
		– » –	+	Brazil	Ferreira et al. 1979
405.	*U. ovipositrix* Calvert, 1909	25(24A+X)	+	Surinam	Kiauta 1979a
		– » –	–	Brazil	Ferreira et al. 1979
406.	***Urothemis****edwardsi* (Selys, 1849)	25(24A+X)	+	Sudan	Wasscher 1985
407.	*U. signata* (Rambur, 1842)	25(24A+X)	+	India	Das 1956 (*U. s. signata* (Rambur, 1842))
		– » –	+	India	Dasgupta 1957 (*U. s. signata*)
		– » –	+	Nepal	Kiauta 1975
		– » –	+	India	Prasad and Thomas 1992
408.	***Zenithoptera****fasciata* (Linnaeus, 1758)	25(24A+X)	+	Surinam	Kiauta 1979a
409.	*Z. lanei* Santos, 1941	25(24A+X)	+	Surinam	Kiauta 1979a
		– » –	+	Brazil	Ferreira et al. 1979
410.	*Z. viola* Ris, 1910	25(24A+X)	+	Bolivia	Cumming 1964
411.	***Zygonyx****iris* Kirby, 1900	23(22A+X)	+	Thailand	Kiauta and Kiauta 1983 (*Z. i. malayanus* (Laidlaw, 1902))
412.	*Z. torrida* (Kirby, 1889)	25(24A+X)	+	India	Tyagi 1978a, b
413.	***Zyxomma****petiolatum* (Rambur, 1842)	25(24A+X)	+	India	Prasad and Thomas 1992
** Cordulegastroidea **					
** Chlorogomphidae **					
414.	***Watanabeopetalia****atkinsoni* (Selys, 1878)	25(24A+X)	+	India	Walia and Chahal 2019
** Cordulegastridae **					
415.	***Anotogaster****basalis* Selys, 1854	23(22A+X)	–	India	Sandhu and Malhotra 1994b
416.	*A. kuchenbeiseri* (Förster, 1899)	25(24A+X)	+	China	Zhu and Wu 1986
417.	*A. sieboldii* (Selis, 1854)	25(24A+X)	+	Japan	Oguma 1930
		– » –	+	Japan	Kichijo 1942a
		– » –	+	Japan	Kiauta 1969a
		– » –	+	Russia	Perepelov et al. 2001
418.	***Cordulegaster****boltoni* (Donovan, 1807)	25(24A+X)	+	Finland	Oksala 1939a, b
		– » –	–	Austria	Kichijo 1942a
		– » –	+	Sweden	Kiauta 1968d, e, 1969a
419.	*C. brevistigma* Selys, 1854	25(24A+X)	+	India	Walia and Chahal 2019
420.	*C. diastatops* (Selys, 1854)	25(24A+X)	+	USA	Cruden 1968
421.	*C. dorsalis* Hagen, 1857	25(24A+X)	+	USA	Cruden 1968
422.	*C. maculata* Selys, 1854	25(24A+X)	+	USA	Cruden 1968
423.	***Neallogaster****hermionae* (Fraser, 1927)	25(24A+X)	+	Nepal	Kiauta and Kiauta 1976
** Zygoptera **					
** Lestoidea **					
** Lestidae **					
424.	***Austrolestes****colensonis* (White, 1846)	25(24A+X)	+	New Zealand	Jensen 1980
425.	***Chalcolestes****viridis* (Van der Linden, 1825)	25(24A+X)	+	Netherlands	Kiauta 1969a
426.	***Indolestes****cyaneus* (Selys, 1862)	25(24A+X)	+	Nepal	Kiauta and Kiauta 1976 as *I. cyanea* (Selys, 1862)
427.	***Lestes****barbarus* (Fabricius, 1798)	25(24A+X)	+	Former Yugoslavia	Kiauta 1972a
428.	*L. congener* Hagen, 1861	25(24A+X)	+	USA	Cruden 1968
429.	*L. disjunctus* Selys, 1862	25(24A+X)	–	USA	Cruden 1968
430.	*L. dorothea* Fraser, 1924	25(24A+X)	+	Nepal	Kiauta 1974, 1975
431.	*L. dryas* Kirby, 1890	25(24A+X)	–	USA	Cruden 1968
		– » –	+	Russia	Perepelov and Bugrov 2001b
432.	*L. forcipatus* Rambur, 1842	21(20A+X)	–	USA	Cruden 1968
433.	*L. forficula* Rambur, 1842	25(24A+X)	+	Jamaica	Cumming 1964
434.	*L. paulistus* Calvert, 1909	25(24A+X)	+	Brazil	Souza Bueno 1982
435.	*L. rectangularis* Say, 1839	25(24A+X)	+	USA	Cruden 1968
436.	*L. similatrix* McLachlan, 1895	25(24A+X)	+	Madagascar	Kiauta 1969b
437.	*L. sponsa* (Hansemann, 1823)	25(24A+X)	–	Former USSR	Makalowskaja 1940
		– » –	+	Japan	Kichijo 1941, 1942a, d, e
		– » –	+	Russia	Perepelov and Bugrov 2001b
438.	*L. stultus* Hagen, 1861	25(24A+X)	+	USA	Cruden 1968
439.	*L. vidua* Hagen, 1861	25(24A+X)	+	USA	Cumming 1964
440.	*L. vigilax* Selys, 1862	19(18A+X)	–	USA	Kiauta and Brink 1978
441.	*L. virens* Charpentier, 1825	25(24A+X)	+	Netherlands	Kiauta 1969a (*L. v. vestalis* Rambur, 1842)
442.	***Sympecma****fusca* (Van der Linden, 1823)	25(24A+X)	+	Japan	Kichijo 1941, 1942d, e
443.	*S. paedisca* (Brauer, 1877)	25(24A+X)	+	Netherlands	Kiauta and Kiauta-Brink 1975 (*S. annulata braueri* (Bianchi, 1904))
		– » –	+	Russia	Perepelov 2003 (*S. a. braueri*)
** Synlestidae **					
444.	***Megalestes****major* Selys, 1862	25(24A+X)	–	Nepal	Kiauta 1974, 1975
** Platystictoidea **					
** Platystictidae **					
445.	***Drepanosticta*** sp.	25(24A+X)	–	Nepal	Kiauta and Kiauta 1976
446.	*Drepanosticta* sp.	25(24A+X)	–	India	Tyagi 1978a, b
447.	***Palaemnema****paulina* (Drury, 1773)	25(24A+X)	+	Costa Rica	Cumming 1964
448.	***Protosticta*** sp.	25(24A+X)	–	Tailand	Kiauta and Kiauta 1983
** Calopterygoidea **					
** Calopterygidae **					
449.	***Atrocalopteryx****atrata* (Selys, 1853)	25(24A+X)	+	Japan	Oguma 1930 as *Calopteryx atrata* Selys, 1853
		– » –	+	Japan	Kichijo 1942d as *C. atrata*
		– » –	+	Japan	Omura 1957 as *C. atrata*
450.	***Calopteryx****aequabilis* Say, 1839	25(24A+X)	+	USA	Cruden 1968
451.	*C. cornelia* (Selys, 1853)	25(24A+X)	+	Japan	Oguma 1930 as *Anaciagrion cornelia* (Selys, 1853)
		– » –	+	Japan	Kichijo 1942a as *A. cornelia*
452.	*C. dimidiata* Burmeister, 1839	25(24A+X)	+	USA	Kiauta and Brink 1978
453.	*C. japonica* Selys, 1869	25(24A+X)	+	Japan	Kichijo 1942a
		– » –	+	Japan	Hirai 1956
		– » –	+	Japan	Omura 1957
		– » –	+	Japan	Kiauta 1968e, f
454.	*C. maculata* (Beauvois, 1805)	25(24A+X)	+	USA	Cumming 1964a
		– » –	+	USA	Cruden 1968
455.	*C. splendens* (Harris, 1780)	25(24A+X)	+	Turkey	Kiauta 1972a (*C. s. amasina* Bartenev, 1912)
455.	*C. splendens* (Harris, 1780)	– » –	+	Italy	Kiauta 1971a (*C. s. caprai* Conci, 1956)
		– » –	–	Former USSR	Makalowskaja 1940 (*C. s. splendens* (Harris, 1782))
		– » –	–	Finland	Oksala 1945 (*C. s. splendens*)
		– » –	–	Germany	Kiauta 1969a, 1971b (*C. s. splendens*)
		– » –	–	France	Kiauta 1973b (*C. s. splendens*)
		– » –	–	Russia	Perepelov et al. 1998 (*C. s. splendens*)
		– » –	+	Russia	Kuznetsova et al. 2020b
456.	*C. virgo* (Linnaeus, 1758)	25(24A+X)	+	Spain	Kiauta 1971b (*C. v. meridionalis* Selys, 1873)
		27(26A+X)	+		
		25(24A+X)	+	Slovenija	Kiauta 1967a, 1968b, c (*C. v. padana* Conci, 1956)
		– » –	+	Austria	Kiauta 1967a, 1968b, c (*C. v. padana*)
		– » –	–	Belgium	Carnoy 1885 (*C. v. virgo* (Linnaeus, 1758))
		– » –	+	Finland	Oksala 1939 (*C. v. virgo*)
		– » –	+	Former USSR	Makalowskaja 1940 (*C. v. virgo*)
		– » –	+	Germany, Luxembourg	Kiauta 1968e, f (*C. v. virgo*)
		– » –	+	Netherlands	Kiauta 1972c (*C. v. virgo*)
		– » –	+	Russia	Kuznetsova et al. 2020b
457.	***Hetaerina****americana* (Fabricius, 1798)	25(24A+X)	+	USA	Cumming 1964
		– » –		USA	Cruden 1968
458.	*H. charca* Calvert, 1909	25(24A+X)	+	Bolivia	Cumming 1964
459.	*H. longipes* (Hagen in Selys, 1853)	25(24A+X)	+	Brazil	Souza Bueno 1982 as *H. carnifex* Hagen in Selys, 1853
		– » –	+	Brazil	Agopian and Mola 1984 as *H. carnifex*
460.	*H. rosea* Selys, 1853	27(26A+X)	+	Bolivia	Cumming 1964
		– » –	+	Bolivia	Kiauta 1969c
		25(24A+X)	–	Brazil	Ferreira et al. 1979
		27(26A+X)	+		
461.	*H. sanguinea* Selys, 1853	25(24A+X)	–	Bolivia	Cumming 1964
462.	*H. titia* (Drury, 1773)	25(24A+X)	+	USA	Cumming 1964
		– » –	+	Mexico	Kiauta 1970a as *H. tricolor* (Burmeister, 1839)
463.	*H. vulnerata* (Selys, 1853)	25(24A+X)	+	Mexico	Kiauta 1970a
464.	***Matrona****basilaris* Selys, 1853	25(24A+X)	–	Taiwan	Kiauta 1968c
465.	***Mnais****costalis* Selys, 1869	25(24A+X)	+	Japan	Oguma 1930
		– » –	+	Japan	Kichijo 1942a
466.	*M. pruinosa* Selys, 1853	25(24A+X)	+	Japan	Oguma 1930 as *M. strigata* Selys, 1853
		– » –	+	Japan	Kichijo 1942a as *M. strigata*
		– » –	+	Japan	Omura 1957 as *M. strigata*
467.	***Neurobasis****chinensis* (Linnaeus, 1758)	23(22A+X)	–	Nepal	Kiauta 1975 (*N. c. chinensis* (Linnaeus, 1758))
		25(24A+X)	–		
		23(22A+X)	–	India	Tyagi 1978b (*N. c. chinensis*)
		– » –	+	Nepal	Kiauta and Kiauta 1982 (*N. c. chinensis*)
		– » –	–	Thailand	Kiauta and Kiauta 1983 (*N. c. chinensis*)
		– » –	+	India	Walia and Sandhu 2002 (*N. c. chinensis*)
		– » –	–	India	Walia et al. 2016 (*N. c. chinensis*)
		– » –	–	India	Walia and Katnoria 2018 (*N. c. chinensis*)
468.	***Phaon****iridipennis* (Burmeister, 1839)	25(24A+X)	+	Republic of South Africa	Boyes et al. 1980
** Chlorocyphidae **					
469.	***Aristocypha****fenestrella* Rambur, 1842	23(22A+X)	–	Thailand	Kiauta and Kiauta 1983 as *Rhinocypha fenestrella* Rambur, 1842
470.	*A. quadrimaculata* (Selys, 1853)	23(22A+X)	+	India	Chatterjee and Kiauta 1973 as *Rhinocypha quadrimaculata* Selys, 1853
		– » –	+	Nepal	Kiauta and Kiauta 1982 as *Rh. quadrimaculata*
471.	*A. trifasciata* (Selys, 1853)	23(22A+X)	–	India	Tyagi 1978a, b as *Rhinocypha trifasciata* Selys, 1853
		– » –	+	Nepal	Kiauta and Kiauta 1982 as *Rh. trifasciata*
472.	***Heliocypha****biforata* (Selys, 1859)	23(22A+X)	–	India	Tyagi 1978a, b as *Rhinocypha biforata beesoni* Selys, 1859
473.	*H. biseriata* (Selys, 1859)	23(22A+X)	–	Thailand	Kiauta and Kiauta 1983 as *Rhinocypha b. biforata* Selys, 1859
474.	***Libellago****lineata* (Burmeister, 1839)	23(22A+X)	–	India	Walia et al. 2018 (*L. l. lineata* (Burmeister, 1839))
		25(24A+X)	–		
475.	***Paracypha****unimaculata* (Selys, 1879)	23(22A+X)	+	Nepal	Kiauta 1974, 1975 as *Rhinocypha unimaculata* Selys, 1879
		– » –	+	Nepal	Kiauta and Kiauta 1982 as *Rh. unimaculata*
476.	***Rhinocypha****colorata* Selys, 1869	23(22A+X)	–	Philippines	Kiauta and Kiauta 1980b
		25(24A+X)	–		
477.	***Vestalis****gracilis* (Rambur, 1842)	25(24A+X)	+	Thailand	Kiauta and Kiauta 1983
** Polythoridae **					
478.	***Cora****irene* Ris, 1918	23(22A+X)	–	Bolivia	Cumming 1964
479.	***Polythore****boliviana* (McLachlan, 1878)	23(22A+X)	–	Bolivia	Cumming 1964
** Euphaeidae **					
480.	***Anisopleura****comes* Hagen, 1880	25(24A+X)	+	Nepal	Kiauta and Kiauta 1976, 1982
481.	***Bayadera****indica* (Selys, 1853)	25(24A+X)	+	Nepal	Chatterjee and Kiauta 1973
		– » –	+	Nepal	Kiauta 1975
482.	***Euphaea****guerini* Rambur, 1842	25(24A+X)	–	Thailand	Kiauta and Kiauta 1983
483.	***Epallage****fatime* (Charpentier, 1840)	25(24A+X)	–	Greece	Kiauta 1970b
		– » –	–	Greece	Chatterjee and Kiauta 1973
** Megapodagrionidae **					
484.	***Allopodagrion****contortum* (Selys, 1862)	25(24A+X)	+	Brazil	Kiauta 1972b as *Megapodagrion contortum* (Selys, 1862)
485.	***Teinopodagrion****macropus* (Selys, 1862)	25(24A+X)	–	Bolivia	Cumming 1964 as *Megapodagrion macropus* (Selys, 1862)
486.	*T. setigerum* (Selys, 1886)	25(24A+X)	–	Bolivia	Cumming 1964 as *Megapodagrion setigerum* Selys, 1886
** Heteragrionidae **					
487.	***Heteragrion****flavidorsum* Calvert, 1909	25(24A+X)	–	Bolivia	Cumming 1964
488.	*H. inca* Calvert, 1909	25(24A+X)	+	Bolivia	Cumming 1964
** Philogeniidae **					
489.	***Philogenia****carrillica* Calvert, 1907	25(24A+X)	+	Costa Rica	Cumming 1964
** Hypolestidae **					
490.	***Hypolestes****clara* (Calvert, 1891)	l7(16A+X)	–	Jamaica	Cumming 1964
** Coenagrionoidea **					
** Platycnemididae **					
491.	***Calicnemia****miniata* (Selys, 1886)	25(24A+X)	+	Nepal	Kiauta and Kiauta 1982
492.	*C. pulverulans* (Selys, 1886)	25(24A+X)	–	Nepal	Kiauta 1975
493.	*Calicnemia* sp.	25(24A+X)	–	Nepal	Kiauta 1975
494.	*Calicnemia* sp.	25(24A+X)	–	India	Tyagi 1978b
495.	***Coeliccia****chromothorax* (Selys, 1891)	25(24A+X)	–	India	Walia and Devi 2020b
496.	*C. bimaculata* (Laidlaw, 1914)	25(24A+X)	–	India	Walia and Devi 2020b
497.	*C. didyma* (Selys, 1863)	25(24A+X)	–	India	Walia and Devi 2020b
498.	*C. fraseri* (Laidlaw, 1932)	25(24A+X)	–	India	Walia and Devi 2020b
499.	*C. renifera* (Selys, 1886)	25(24A+X)	–	Nepal	Kiauta 1974, 1975
		– » –	–	India	Walia and Devi 2020b
500.	***Copera****annulata* (Selys, 1863)	25(24A+X)	+	Japan	Kichijo 1941, 1942a, c
		– » –	+	India	Dasgupta 1957
		– » –	–	Thailand	Kiauta and Kiauta 1983
		– » –	+	India	Walia and Devi 2018
501.	*C. marginipes* (Rambur, 1842)	25(24A+X)	–	India	Tyagi 1978a, b
		– » –	–	Thailand	Kiauta and Kiauta 1983
		– » –	+	India	Walia and Devi 2018
502.	*C. vittata* (Selys, 1863)	25(24A+X)	+	India	Walia and Devi 2018
		– » –	+	India	Walia and Devi 2018 (*C. v. assamensis* (Laidlaw, 1914))
503.	***Disparoneura****quadrimaculata* (Rambur, 1842)	25(24A+X)	–	India	Walia and Devi 2020a
504.	***Esme****cyaneovittata* Fraser, 1922	25(24A+X)	–	India	Walia and Devi 2020a
505.	*E. longistyla* Fraser, 1931	25(24A+X)	–	India	Walia and Devi 2020a
506.	***Onychargia****atrocyana* (Selys, 1865)	25(24A+X)	–	Thailand	Kiauta and Kiauta 1983
507.	***Platycnemis****pennipes* (Pallas, 1771)	25(24A+X)	–	Finland	Oksala 1945
		– » –	–	Italy	Kiauta 1971a
		– » –	–	Russia	Perepelov and Bugrov 2001b
508.	***Prodasineura****autumnalis* (Fraser, 1922)	25(24A+X)	+	Thailand	Kiauta and Kiauta 1983
509.	*P. nigra* (Fraser, 1922)	25(24A+X)	–	India	Walia and Devi 2020a
510.	*P. verticalis* (Selys, 1860)	25(24A+X)	–	India	Walia and Devi 2020a
511.	*Prodasineura* sp.1	25(24A+X)	–	Thailand	Kiauta and Kiauta 1983
512.	*Prodasineura* sp.2	25(24A+X)	–	Thailand	Kiauta and Kiauta 1983
** Coenagrionidae **					
513.	***Acanthagrion****ascendens* Calvert, 1909	27(26A+X)	+	Bolivia	Cumming 1964
514.	*A. chacoense* Calvert, 1909	27(26A+X)	+	Bolivia	Cumming 1964
515.	*A. gracile* (Rambur, 1842)	27(26A+X)	–	Surinam	Kiauta 1979a (*A. g. minarum* Selys, 1876)
		– » –	–	Brazil	Ferreira et al. 1979 (*A. g. minarum* Selys, 1876)
516.	***Aeolagrion***inca Selys, 1876	27(26A+X)	–	Bolivia	Cumming 1964 as *A. foliaceum* (Sjöstedt, 1918)
517.	***Agriocnemis****clauseni* Fraser, 1922	27(26A+X)	+	India	Tyagi 1978a, b
518.	*A. femina* (Brauer, 1868)	27(26A+X)	–	Philippines	Kiauta and Kiauta 1980b
		– » –	+	Thailand	Kiauta and Kiauta 1983
519.	*A. pygmaea* (Rambur, 1842)	27(26A+X)	–	India	Tyagi 1978b
		– » –	+	Thailand	Kiauta and Kiauta 1983
520.	***Amphiagrion****abbreviatum* (Selys, 1876)	27(26A+X)	–	USA	Cruden 1968
521.	***Amphiallagma****parvum* (Selys, 1876)	27(26A+X)	+	India	Handa and Kochhar 1985 as *Enallagma parvum* Selys, 1876
522.	***Argia****apicalis* (Say, 1839)	37(36A+X)	–	USA	Kiauta and Kiauta 1980b
523.	*A. fumipennis* (Burmeister, 1839)	27(26A+X)	–	USA	Kiauta and Kiauta 1980c (*A. f. atra* Gloyd, 1968)
		– » –	–	USA	Kiauta and Brink 1978 (*A. f. fumipennis* (Burmeister, 1839))
		– » –	–	USA	Kiauta and Kiauta 1980c (*A. f. fumipennis*)
		– » –	+	Canada	Kiauta and Kiauta 1980c (*A. f. violacea* (Hagen, 1861))
524.	*A. funebris* (Hagen, 1861)	27(26A+X)	–	USA	Kiauta 1972b
		28(26A+XX)*	–	Mexico	Kiauta and Kiauta 1980c
525.	*A. immunda* (Hagen, 1861)	27(26A+X)	–	USA	Kiauta and Kiauta 1980c
526.	*A. moesta* (Hagen, 1861)	25(24A+X)	–	Canada	Kiauta 1978
		– » –	–	USA	Kiauta and Kiauta 1980c
527.	*A. nahuana* Calvert, 1902	25(24A+X)	–	USA	Kiauta and Kiauta 1980c
528.	*A. sedula* (Hagen, 1861)	27(26A+X)	–	USA	Cruden 1968
		– » –	–	USA	Kiauta and Kiauta 1980c
529.	*A. tibialis* (Rambur, 1842)	37(36A+X)	–	USA	Kiauta and Kiauta 1980c
530.	*A. translata* Hagen, 1865	25(24A+X)	+	USA	Kiauta and Kiauta 1980c
531.	*A. violacea* (Hagen, 1861)	27(26A+X)	–	USA	Cruden 1968
532.	*A. vivida* (Hagen, 1861)	27(26A+X)	–	USA	Cruden 1968
533.	***Ceriagrion****auranticum* Fraser, 1922	27(26A+X)	+	Thailand	Kiauta and Kiauta 1983 as *C. latericium* Lieftinck, 1951
534.	*C. azureum* (Selys, 1891)	27(26A+X)	–	Nepal	Kiauta 1974, 1975
535.	*C. cerinomelas* Lieftinck, 1927	27(26A+X)	–	Nepal	Kiauta 1974, 1975
536.	*C. cerinorubellum* (Brauer, 1866)	27(26A+X)	+	India	Dasgupta 1957
		– » –	+	India	Prasad and Thomas 1992
537.	*C. coromandelianum* (Fabricius, 1798)	27(26A+X)	+	India	Ray Chaudhuri and Dasgupta 1949
		– » –	+	India	Srivastava and Das 1953
		– » –	+	India	Das 1956
		– » –	+	Nepal	Kiauta and Kiauta 1982
		– » –	+	India	Prasad and Thomas 1992
538.	*C. fallax* Ris, 1914	27(26A+X)	+	Republic of South Africa	Dasgupta 1957
539.	*C. glabrum* (Burmeister, 1839)	27(26A+X)	–	Kingdom of Eswatini (Former Swaziland)	Boyes et al. 1980
540.	*C. rubiae* Laidlaw, 1916	27(26A+X)	+	India	Asana and Makino 1935
		– » –	+	India	Makino 1935
		– » –	+	India	Kichijo 1942a
541.	*C. tenellum* (Villers, 1789)	27(26A+X)	+	Italy	Kiauta 1971a (*C. t. tenellum* (Villers, 1789))
542.	***Chromagrion****conditum* (Hagen, 1876)	27(26A+X)	–	USA	Cruden 1968
543.	***Coenagrion****armatum* (Charpentier, 1840)	27(26A+X)	–	Finland	Oksala 1939a
		– » –	–	Former USSR	Makalowskaja 1940
544.	*C. hastulatum* (Charpentier, 1825)	27(26A+X)	–	Former USSR	Makalowskaja 1940
		– » –	–	Russia	Perepelov and Bugrov 2001b
545.	*C. hylas* (Trybom, 1889)	27(26A+X)	–	Austria	Kiauta and Kiauta 1991 (*C. h. freyi* (Bilek, 1954))
546.	*C. lunulatum* (Charpentier, 1840)	27(26A+X)	+	Russia	Perepelov and Bugrov 2001b
547.	*C. pulchellum* (Vander Linden, 1823)	27(26A+X)	–	Former USSR	Makalowskaja 1940
		– » –	–	Netherlands	Kiauta 1969c
		– » –	+	Russia	Kuznetsova et al. 2020b
548.	*C. puella* (Linnaeus, 1758)	27(26A+X)	+	Russia	Kuznetsova et al. 2020b
549.	*C. resolutum* (Hagen, 1876)	27(26A+X)	–	USA	Cruden 1968
550.	*Coenagrion* sp.	27(26A+X)	+	Japan	Kichijo 1941, 1942d, e
551.	***Diceratobasis****macrogaster* (Selys, 1875)	27(26A+X)	+	Jamaica	Cumming 1964
552.	***Enallagma****aspersum* (Hagen, 1861)	27(26A+X)	–	USA	Cruden 1968
553.	*E. boreale* Selys, 1875	27(26A+X)	–	USA	Cruden 1968
554.	*E. carunculatum* Morse, 1895	27(26A+X)	–	USA	Cruden 1968
555.	*E. circulatum* Selys, 1883	27(26A+X)	+	Russia	Perepelov and Bugrov 2001b
556.	*E. civile* (Hagen, 1861)	27(26A+X)	–	USA	Cruden 1968
557.	*E. cyathigerum* (Charpentier, 1840)	27(26A+X)	–	Finland	Oksala 1939a, 1945
		– » –	–	Former USSR	Makalowskaja 1940
		– » –	+	USA	Brink and Kiauta 1964
		27(26A+X),	–	USA	Cruden 1968
		29(28A+X)	–		
		27(26A+X)	+	Netherlands	Kiauta 1969a, c
		29(28A+X)	+		
558.	*E. ebrium* (Hagen, 1861)	27(26A+X)	–	USA	Cruden 1968
559.	*E. praevarum* (Hagen, 1861)	27(26A+X)	–	USA	Cruden 1968
560.	***Erythromma****lindeni* (Selys, 1840)	27(26A+X)	+	Italy	Kiauta 1971a
561.	*E. najas* (Hansemann, 1823)	27(26A+X)	–	Finland	Oksala 1939a
		– » –	–	Former USSR	Makalowskaja 1940
		– » –	–	Netherlands	Kiauta 1969a
		– » –	–	Russia	Perepelov and Bugrov 2001b
		– » –	+	Russia	Kuznetsova et al. 2020b
562.	***Homeoura****chelifera* (Selys, 1876)	27(26A+X)	+	Surinam	Kiauta 1979a as *Enallagma cheliferum* (Selys, 1876)
		– » –	+	Brazil	Ferreira et al. 1979 as *E. cheliferum*
563.	***Ischnura****aurora* (Brauer, 1865)	27(26A+X)	–	Nepal	Kiauta 1974, 1975
		– » –	–	India	Handa and Kochhar 1985
564.	*I. capreola* (Hagen, 1861)	27(26A+X)	–	Bolivia	Cumming 1964 as *Ceratura capreola* (Hagen, 1861)
565.	*I. cervula* Selys, 1876	27(26A+X)	–	USA	Cruden 1968
566.	*I. denticollis* (Burmeister, 1839)	27(26A+X)	–	USA	Cruden 1968
567.	*I. elegans* (Van der Linden, 1823)	27(26A+X)	–	Finland	Oksala 1939a, 1945
		– » –	–	Netherlands	Kiauta 1969a
		– » –	–	Russia	Perepelov 2003
568.	*I. fluviatilis* Selys, 1876	27(26A+X)	–	Bolivia	Cumming 1964
569.	*I. forcipata* Morton, 1907	27(26A+X)	–	Nepal	Kiauta 1974, 1975
570.	*I. nursei* (Morton, 1907)	25(24A+X)	+	India	Tyagi 1978b as *Rhodischnura nursei* (Morton, 1907)
571.	*I. pumilio* (Charpentier, 1825)	27(26A+X)	+	Netherlands	Kiauta 1979b
572.	*I. perparva* Selys, 1876	27(26A+X)	–	USA	Cruden 1968
573.	*I. ramburii* (Selys, 1850)	27(26A+X)	+	USA	Kiauta and Brink 1978
574.	*I. rufostigma* Selys, 1876	27(26A+X)	–	Nepal	Kiauta 1974, 1975 (*I. r. annandalei* Laidlaw, 1919)
575.	*I. senegalensis* (Rambur, 1842)	27(26A+X)	+	Japan	Kichijo 1941, 1942d, e
		– » –	+	India	Dasgupta 1957
		– » –	+	Ethiopia	Kiauta 1969b
		– » –	+	Philippines	Kiauta and Kiauta 1980b
		– » –	–	Thailand	Kiauta and Kiauta 1983
		– » –	+	India	Prasad and Thomas 1992
576.	*I. verticalis* (Say, 1839)	27(26A+X)	–	USA	Cruden 1968
577.	*I. ultima* Ris, 1908	27(26A+X)	–	Bolivia	Cumming 1964
578.	***Leptagrion****macrurum* (Burmeister, 1839)	30(28A+neo-XY)	–	Brazil	Kiauta 1971c, 1972d
579.	***Mecistogaster***. sp. 1	29(28A+X)	+	Bolivia	Cumming 1964
580.	*Mecistogaster* sp. 2	12(10A+neo-XY)	–	Bolivia	Cumming 1964
581.	***Megalagrion****oahuense* (Blackburn, 1884)	27(26A+X)	+	Hawaii	Kiauta 1969b
582.	***Mortonagrion****selenion* (Ris, 1916)	27(26A+X)	+	Japan	Kichijo 1941, 1942a, d, e
583.	***Nehalennia****irene* (Hagen, 1861)	27(26A+X)	–	USA	Cruden 1968
584.	*N. speciosa* (Charpentier, 1840)	28(26A+XX)*	–	Finland	Oksala 1945
585.	***Oxyagrion****hempeli* Calvert, 1909	27(26A+X)	–	Brazil	Souza Bueno 1982
586.	*O. terminale* Selys, 1876	27(26A+X)	–	Surinam	Kiauta 1979a
		– » –	–	Brazil	Ferreira et al. 1979
587.	***Paracercion****hieroglyphicum* (Brauer, 1865)	27(26A+X)	+	Japan	Kichijo 1941, 1942d, e as *Coenagrion hieroglyphicum* (Brauer, 1865)
588.	*P. malayanum* (Selys, 1876)	27(26A+X)	+	Nepal	Kiauta 1974, 1975
589.	***Proischnura****subfurcata* (Selys, 1876)	27(26A+X)	–	Kenya	Wasscher 1985 as *Enallagma subfurcatum* Selys, 1876
590.	***Pseudagrion****acaciae* Förster, 1906	27(26A+X)	+	Republic of South Africa	Boyes et al. 1980
591.	*P. australasiae* Selys, 1876	27(26A+X)	+	India	Dasgupta 1957
592.	*P. decorum* (Rambur, 1842)	27(26A+X)	+	India	Dasgupta 1957
593.	*P. kersteni* (Gerstaker, 1869)	27(26A+X)	–	Kingdom of Eswatini (Former Swaziland)	Boyes et al. 1980
594.	*P. microcephalum* (Rambur, 1842)	27(26A+X)	+	India	Dasgupta 1957
		– » –	+	Philippines	Kiauta and Kiauta 1980b
595.	*P. pruinosum* (Burmeister, 1839)	27(26A+X)	+	Thailand	Kiauta and Kiauta 1983
596.	*P. rubripes* (Selys, 1876)	27(26A+X)	+	India	Dasgupta 1957
		– » –	+	Philippines	Kiauta and Kiauta 1980b
		– » –	+	Thailand	Kiauta and Kiauta 1983
597.	*P. salisburyense* Ris, 1921	27(26A+X)	+	Kingdom of Eswatini (Former Swaziland)	Boyes et al. 1980
598.	*P. spencei* Fraser, 1922	27(26A+X)	+	India	Dasgupta 1957
599.	*P. whellani* Pinhey, 1956	25(24A+X)	+	Burkina Faso (Former Voltiac Republic)	Kiauta and Ochssée 1979
600.	***Pyrrhosoma****nymphula* (Sutzer, 1776)	28(26A+XX)*	–	Finland	Oksala 1945
601.	***Telebasis****carmesina* Calvert, 1909	27(26A+X)	–	Surinam	Kiauta 1979a
		– » –	–	Brazil	Ferreira et al. 1979
602.	***Tigriagrion****aurantinigrum* Calvert, 1909	27(26A+X)	–	Bolivia	Cumming 1964
603.	***Xanthocnemis****zealandica* (McLachlan, 1873)	27(26A+X)	–	New Zealand	Jensen 1980 as *X. zelandica* (McLachlan, 1873)
604.	***Zoniagrion****exclamationis* (Selys, 1876)	27(26A+X)	–	USA	Cruden 1968
** Protoneuridae **					
605.	***Caconeura****autumnalis* Fraser, 1922	25(24A+X)	+	India	Tyagi 1978b
606.	***Epipleoneura*** sp.	27(26A+X)	–	Bolivia	Cumming 1964
607.	***Protoneura****rubriventris* (Selys, 1860)	27(26A+X)	+	Bolivia	Cumming 1964 as *Neoneura rubriventris* Selys, 1860

* In the original publication, the female karyotype is given. ** Jensen (1980) considers these data as erroneous (but see section “Concluding remarks and future directions” in the present paper). *** Karyotype formula is extrapolated based on vague descriptions by Cumming (1964).

Within Odonata, chromosome numbers in males vary over a relatively wide range, from 2n = 6 in *Macrothemis hemichlora* and *Orthemis levis* to 2n = 41 in *O. nodiplaga*. Both low chromosome number species are suggested to have an evolutionarily secondary neo-XY system (Cumming 1964; Kiauta 1972c) that could have arisen through an X-autosome fusion from an X(0) system. All three of the above species belong to the largest dragonfly family Libellulidae, in which nearly 89% of studied species (255 in total) have the karyotype 2n = 25(24A + X). The last one is the most common in Odonata in general: it occurs in each of the three suborders, Zygoptera, Anisoptera and Anisozygoptera, and in all families with the exception of two damselfly families, the Polythoridae with only two studied species sharing 2n = 23(22A + X) and a monotypic family Hypolestidae with 2n = 17(16A + X) in male *Hypolestes clara*. Besides Libellulidae, the karyotype 2n = 25(24A + X) is currently the presumed modal one in 14 other families, such being the case at least in six better covered (at species and/or generic level) families, i.e. the dragonfly families Corduliidae, Cordulegastridae, and Macromiidae, and the damselfly families Lestidae, Calopterygidae, and Platycnemididae (Table 2, Fig. 1). This chromosome set is suggested to be an ancestral one for the order Odonata in general (Oguma 1930; Kuznetsova et al. 2020b) although this suggestion remains questionable at this stage.

**Table 2.** The diversity of chromosome numbers and sex chromosome mechanisms, and modal karyotypes in 23 families of Odonata: a summary.

Taxa		N of species/ genera studied	Male karyotypes	Modal karyotype	N of species/genera with modal karyotype (occurrence in percent)
(N of species/genera described*)					
Anisozygoptera					
Epiophlebioidea	Epiophlebiidae (4/1)	1/1	25, X0	24A + X	1 (100) / 1 (100)
Anisoptera					
Aeshnoidea	Aeshnidae (456/51)	58/18	13, X0; 14, neo-XY; 15, X0; 16, neo-XY; 19, X0; 21, X0; 24, neo-XY; 25, X0; 26, neo-XY; 27, X0	26A + X	44 (76) / 14 (78)
Petaluroidea	Petaluridae (10/5)	4/3	17, X0; 19, X0; 25, X0	16A + X	3 (75) / 2 (67)
Gomphoidea	Gomphidae (980/87)	66/31	12, neo-neo-XY; 21, X0; 22, neo-XY; 23, X0; 24, neo-XY; 25, X0	22A + X	57 (86) / 28 (90)
Libelluloidea	Macromiidae (125/4)	6/3	25, X0	24A + X	6 (100) / 3 (100)
	Corduliidae (154/20)	23/7	10, neo-XY; 11, X0; 13, X0; 14, neo-XY, 20, XY; 21, X0; 25, X0; 26, neo-XY; 27, X0	24A + X	19 (83) / 6 (86)
	Libellulidae (1037/142)	255/59	6, neo-XY; 6 neo-XY; 8, neo-XY; 10, neo-XY; 12, neo-XY; 17, X0; 21, X0; 22, neo-XY; 23, X0; 23, X1X2Y; 24, neo-XY; 25, X0; 27, X0; 28, neo-XY; 29, X0; 41, X0	24A + X	227 (89) / 57 (97)
Cordulegastroidea	Cordulegastridae (46/3)	9/3	23, X0; 25, X0	24A + X	8 (89) / 3 (100)
	Chlorogomphidae (47/3)	1/1	25, X0	24A + X	1 (100) / 1 (100)
Zygoptera					
Lestoidea	Lestidae (151/9)	20/5	19, X0; 21, X0; 25, X0	24A + X	18 (90) / 5 (100)
	Synlestidae (39/9)	1/1	25, X0	24A + X	1 (100) / 1 (100)
Platystictoidea	Platystictidae (224/6)	4/3	25, X0	24A + X	4 (100) / 3 (100)
Calopterygoidea	Calopterygidae (185/21)	20/8	23, X0; 25, X0; 27, X0	24A + X	20 (100) / 8 (100)
	Chlorocyphidae (144/19)	9/6	23, X0; 25, X0	22A + X	8 (89) / 5 (84)
	Polythoridae (59/7)	2/2	23, X0	22A + X	2 (100) / 2 (100)
	Euphaeidae (68/12)	4/4	25, X0	24A + X	4 (100) / 4 (100)
	Megapodagrionidae (296/42)	3/2	25, X0	24A + X	3 (100) / 2 (100)
	Heteragrionidae (57/2)	2/1	25, X0	24A + X	2 (100) / 1 (100)
	Philogeniidae (40/2)	1/1	25, X0	24A + X	1 (100) / 1 (100)
	Hypolestidae (6/4)	1/1	17, X0	16A + X	1 (100) / 1 (100)
Coenagrionoidea	Platycnemididae (404/40)	22/8	25, X0	24A + X	19 (100) / 7 (100)
	Coenagrionidae (1267/114)	92/28	12, neo-XY; 25, X0; 27, X0; 29, X0; 30, neo-XY; 37, X0	26A + X	81 (89) / 26 (90)
	Protoneuridae (260 / 25)	3/3	25, X0; 27, X0	26A + X	2 (70) / 2 (70)

*Taken from Dijkstra et al. 2013

Chromosomal rearrangements, among which fission and fusions apparently predominated (Kiauta 1969c, 1972c), led to the appearance of divergent karyotypes in the evolution of Odonata. As a result, in many dragonfly and damselfly families, other karyotypes, when occurring, are of secondary origin as indicated by either a diverged number of autosomes or a secondary sex chromosome system of an XY-type or both (e.g. Cumming 1964; Kiauta 1969a, c; Agopian and Mola 1984, 1988; Mola et al. 1999; Perepelov and Bugrov 2002). Some interesting examples of this kind can be found in the family Libellulidae, in which 2n = 25(24A + X) is most likely an evolutionarily initial karyotype (e.g. Agopian and Mola 1988). These examples are as follows (see Table 1): *Orthemis nodiplaga* and *O. ambinigra* with 2n = 41(40A + X) and 2n = 12(10A + neo-XY), respectively; *Erythrodiplax media* and *E. minuscula*, both with 2n = 22(20A + neo-XY); *Micrathyria longifasciata* and *M. ungulata* with 2n = 24(22A + neo-XY) and 2n = 23(20A + X_1_X_2_Y), respectively. In some families, any of these presumably derived karyotypes not only occurs but also prevails and may be considered modal (see Table 2 and Fig. 1). Within Anisoptera, such families are Aeshnidae (2n = 26A + X) and Gomphidae (2n = 22A + X), whereas within Zygoptera, these are Chlorocyphidae (2n = 22A + X) and Coenagrionidae (2n = 26A + X). Thus, Odonata, despite the fact that they have holokinetic chromosomes (Nokkala et al. 2002), demonstrate rather high karyotypic stability, with most species showing 2n = 25 (found in 60% of studied species), 2n = 27(21%) and 2n = 23(13%) which may point to some selective constraints acting to stabilize chromosome number in their evolution (Kuznetsova et al. 2020b).

There are the species for which different authors give various karyotypes that are sometimes difficult to interpret (see Table 1). In some cases, this might be due to misidentifications of a particular species or an error in determining the karyotype. For example, Wolfe (1953) reported 2n = 17(16A + X) for males of *Uropetala carovei* (Petaluridae, Anisoptera) from New Zealand. However, according to later studies of this species in the same locality (Jensen and Mahanty 1978; Jensen 1980), it has 2n = 25(24A + X), and Jensen (1980) therefore considers the Wolfe data as erroneous. We cannot exclude, however, that the above authors studied different *U. carovei* subspecies, *U. c. carovei* White, 1846 and *U. c. chiltoni* Tillyard, 1921, that may indeed have different karyotypes. In other cases, the chromosome number difference between geographic populations might be indicative of the inter-population variation within the bounds of one taxonomic species or even the existence of a species complex with several morphologically cryptic species. For example, 4 of the 17 studied species of the dragonfly genus *Aeshna* Fabricius, 1775 were reported to have different karyotypes in different populations. These are: *Aeshna grandis* – 2n = 26A + X (former USSR), 2n = 24A + X (former USSR, Finland), and 2n = 24A + neo-XY (Netherlands, Finland); *A. isoceles* – 2n = 26A + X (USA) and 2n = 24A + X (Russia); *A. juncea* – 2n = 26A + X (Italy) and 2n = 24A + neo-XY (Finland, former USSR, Italy); *A. mixta* – 2n = 26A + X (Netherlands) and 2n = 24A + X (India) (Table 1). In all such cases, special studies involving a combined analysis of karyotypes, morphology, distribution patterns and molecular markers are needed.

Approximately 80% of Odonata species have a pair of very small chromosomes, i.e. microchromosomes or m-chromosomes (Mola 2007, Table 1). A number of speculations have been forwarded to explain the origin of these chromosomes in Odonata. Kiauta (1968e) suggested m-chromosomes to be fragments of “normal” chromosomes, whereas Oguma (1930) considered them the remnants of an autosome pair in the process of its elimination by progressive loss of chromatin. The size of the smaller chromosome pair was shown to be variable within different species (Kiauta 1968e; see Mola 2007 for other references) which is consistent with both hypotheses. Closely related species and different populations of the same species often differ from each other in the presence/absence of m-chromosomes (Table 1). This is most likely due to the lack of clear criteria for the identification of a small chromosome pair as m-chromosomes in a particular karyotype (Mola 2007; Kuznetsova et al. 2020b).

Most cytogenetic studies of Odonata have been made only to determine the chromosome number and sex chromosome mechanism for which the routine staining was used. Although a considerable amount of such data was obtained (Table 1, 2), standard karyotypes of many Odonata taxa remain totally unknown (Fig. 1). Lack of data on more “primitive” families of Zygoptera (e.g. Hemiphlebiidae) and Anisoptera (e.g. Austropetaliidae and Neopetaliidae) makes difficult understanding karyotype evolution of the order in general.

During the last decades, karyotypes of a few dozen Odonata species were studied using various techniques of differential staining of chromosomes such as C-banding, AgNOR-staining and DNA specific fluorochrome banding visualiszing constitutive heterochromatin, nucleolus organizing regions (NORs) and AT- and GC-rich chromosome segments, respectively. Such data can be found in the following publications: Thomas and Prasad (1986), Prasad and Thomas (1992), Perepelov et al. (1998), Perepelov and Bugrov (2001a, b, 2002), Grozeva and Marinov (2007), De Gennaro et al. (2008), Walia et al. (2011, 2018), Walia and Chahal (2014, 2018), Walia and Devi (2018), Walia and Katnoria (2018), Walia and Devi (2020a, b). Unfortunately, these data alone did not shed much light on the karyotypic evolution of Odonata.

Although the classical cytological techniques remain necessary starting points for cytogenetic studies of Odonata to get an overview of their genomes, the future of Odonata cytogenetics must be coupled with the application of new cytogenetic molecular techniques that enable the localization of specific DNA sequences in chromosomes and the identification of individual chromosomes in karyotypes. In the article by Frydrychová et al. (2004) and, on a larger scale, in two of our recent publications (Kuznetsova et al. 2018, 2020b), the fluorescence *in situ* hybridization (FISH) technique was used for the first time for analyzing Odonata karyotypes. Several species belonging to the Anisoptera (from the families Aeshnidae, Libellulidae, and Corduliidae) and the Zygoptera (from the families Coenagrionidae and Calopterygidae) were studied regarding the occurrence of the TTAGG telomeric repeats and the distribution of the *18S rRNA* genes in their karyotypes. The TTAGG repeats proved to be the canonical motif of telomeres in the class Insecta in general, which, however, was repeatedly lost in the evolution of different phylogenetic lineages (Kuznetsova et al. 2020a). It was shown in the listed Odonata publications that the (TTAGG)*_n_* motif does not occur in all but one (*Sympetrum vulgatum*) species, and the *18S* is located on one of the largest pairs of autosomes in all studied dragonfly species but on m-chromosomes in all studied damselfly species (Kuznetsova et al. 2020b).

The results obtained showed great promise of the combined use of FISH and classical and banding cytogenetics in order to identify new chromosomal markers, reveal differences between species, particularly when they share the same or very close karyotypes, and speculate about the mechanisms involved in the karyotype evolution of Odonata (Kuznetsova et al. 2020b). Another promising line of future research could be to test hypotheses (Mola and Papeschi 1994; Ardila-Garcia and Gregory 2009) about whether there is a relationship between karyotype evolution and genome size diversity in the Odonata or there is no such relationship.
